# Concurrent and Longitudinal Contribution of Exposure to Bullying in Childhood to Mental Health: The Role of Vulnerability and Resilience

**DOI:** 10.1001/jamapsychiatry.2017.2678

**Published:** 2017-11-01

**Authors:** Timothy Singham, Essi Viding, Tabea Schoeler, Louise Arseneault, Angelica Ronald, Charlotte M. Cecil, Eamon McCrory, Frülhing Rijsdijk, Jean-Baptiste Pingault

**Affiliations:** 1 Division of Psychology and Language Sciences, University College London, London, England; 2 Social Genetic and Developmental Psychiatry Centre, King’s College London, London, England; 3 Department of Psychological Sciences, University of Birkbeck, London, England; 4 Department of Psychology, King’s College London, London, England

## Abstract

**Importance:**

Exposure to bullying is associated with poor mental health. However, the degree to which observed associations reflect direct detrimental contributions of exposure to bullying to mental health remains uncertain, as noncausal relationships may arise from genetic and environmental confounding (eg, preexisting vulnerabilities). Determining to what extent exposure to bullying contributes to mental health is an important concern, with implications for primary and secondary interventions.

**Objective:**

To characterize the concurrent and longitudinal contribution of exposure to bullying to mental health in childhood and adolescence using a twin differences design to strengthen causal inference.

**Design, Setting, and Participants:**

Participants were drawn from the Twins Early Development Study, a population-based cohort recruited from population records of births in England and Wales between January 1, 1994, and December 31, 1996. Data collection took place when the participants were between 11 and 16 years of age from December 1, 2005, to January 31, 2013. Data analysis was conducted from January 1, 2016, to June 20, 2017.

**Exposures:**

Participants completed the Multidimensional Peer-Victimization Scale at 11 and 14 years of age.

**Main Outcomes and Measures:**

Mental health assessments at 11 and 16 years of age included anxiety, depression, hyperactivity and impulsivity, inattention, conduct problems, and psychotic-like experiences (eg, paranoid thoughts or cognitive disorganization).

**Results:**

The 11 108 twins included in the final sample (5894 girls and 5214 boys) were a mean age of 11.3 years at the first assessment and 16.3 years at the last assessment. The most stringent twin differences estimates (monozygotic) were consistent with causal contribution of exposure to bullying at 11 years to concurrent anxiety, depression, hyperactivity and impulsivity, inattention, and conduct problems. Effects decreased over time; that is, substantial concurrent contributions to anxiety (β = 0.27; 95% CI, 0.22-0.33) persisted for 2 years (β* = *0.12; 95% CI, 0.04-0.20) but not 5 years. Direct contributions to paranoid thoughts and cognitive disorganization persisted for 5 years.

**Conclusions and Relevance:**

This study is the largest to date to characterize the contribution of exposure to bullying in childhood to mental health using a twin differences design and multi-informant, multiscale data. Stringent evidence of the direct detrimental contribution of exposure to bullying in childhood to mental health is provided. Findings also suggest that childhood exposure to bullying may partly be viewed as a symptom of preexisting vulnerabilities. Finally, the dissipation of effects over time for many outcomes highlights the potential for resilience in children who were bullied. In addition to programs that aim to reduce exposure to bullying, interventions may benefit from addressing preexisting vulnerabilities and focus on resilience.

## Key Points

**Question:**

What is the concurrent and longitudinal contribution of exposure to bullying in childhood to mental health?

**Findings:**

This population-based cohort study using a twin differences design (11 108 twins) provides evidence that childhood exposure to bullying directly contributes to multiple mental health domains, particularly anxiety, depression, paranoid thoughts, and cognitive disorganization. This direct contribution dissipates or is reduced after 5 years.

**Meaning:**

In addition to primary prevention aiming to stop childhood exposure to bullying, secondary preventive interventions should support resilience processes and address prior vulnerabilities in children exposed to bullying.

## Teaser

This population-based cohort study uses data from the Twins Early Development Study to characterize the concurrent and longitudinal contribution of exposure to bullying to mental health in childhood and adolescence.

## Introduction

One-third of children report having been bullied by their peers according to estimates from the World Health Organization.^1^ *Childhood exposure to bullying* refers to the experience of being a target of hostile behavior from other children (eg, being physically or verbally attacked)^2^; it is associated with a wide range of long-lasting adverse outcomes, particularly mental health outcomes such as anxiety.^3^ A key challenge for current research is to probe the causal nature of these widespread associations.

Whether cross-sectional or longitudinal, most available studies remain correlational and fall short of being able to infer causality. In particular, most studies are not genetically informative and do not account for genetic confounding. This issue is problematic given that genetic influences account for up to two-thirds of the variation in exposure to bullying, suggesting that being bullied is influenced by preexisting heritable individual vulnerabilities.^6^ For example, prior mental health difficulties, personality, or cognitive deficits may increase the likelihood of being bullied.^7^ The same set of vulnerabilities may also confer an increased risk of developing adverse mental health outcomes later in life. Such person-environment correlations between individual vulnerabilities and exposure to bullying—or gene-environment correlation when driven by genetic factors^10^—can generate associations that do not entirely reflect a causal contribution of childhood exposure to bullying.

To establish causality, experimental designs randomly allocating children to different degrees of exposure to bullying are clearly precluded for ethical reasons. The strongest remaining design is an observational approach based on the counterfactual framework for causal inference.^11^ The counterfactual framework stipulates that, to assess the effect of being exposed to a risk factor (eg, exposure to bullying), an exposed individual should ideally be matched with his or her nonexposed self. Because exposed individuals are the same as control individuals in this ideal scenario, all possible sources of genetic and environmental confounding are controlled for. Naturally, an individual cannot be exposed and not exposed to a risk factor at the same time. Therefore, causal inference methods aim to approximate this ideal scenario. One such powerful method is the twin differences design, in which one twin is used as a control for the other, thereby accounting for shared environmental and genetic sources of confounding, in part for dizygotic (DZ) twins and fully for monozygotic (MZ) twins.

Because of small sample sizes, twin studies on childhood exposure to bullying have not fully implemented this co-twin design (which requires obtaining separate DZ and MZ estimates) except for 2 studies.^13^ Arseneault et al^13^ found that MZ twins who experienced being bullied between the ages of 7 and 9 years (assessed by mothers at age 10 years) had significantly more internalizing problems at age 10 years than did their co-twin. Recently, Silberg et al^14^ examined the contribution of being bullied by peers in childhood to psychiatric disorders in childhood and young adulthood. In MZ analyses, significant concurrent contributions of exposure to bullying were found for anxiety and attention-deficit/hyperactivity disorder in childhood and for suicidal ideation in young adulthood (but not in childhood). Owing to sample size, these analyses were conducted only on a subset of available psychiatric outcomes. In addition, contrasting concurrent vs long-term contributions of childhood exposure to bullying was not possible for most outcomes. Finally, binary measures of bullying across both studies limited power and the ability to study different dimensions of childhood exposure to bullying.

To our knowledge, our study is the largest prospective study to date to use a stringent, genetically informative design to test the degree to which childhood exposure to bullying contributes to mental health difficulties and test whether direct contributions of exposure to bullying persist over time. To this end, we used a multidimensional measure of childhood exposure to bullying assessing different forms of bullying (physical, verbal, social, and property-related) as well as comprehensive multi-informant, multiscale assessments of mental health. Outcomes included anxiety, depression, hyperactivity and impulsivity, inattention, conduct problems, and psychotic-like experiences.

## Methods

### Participants

Participants were drawn from the Twins Early Development Study (TEDS) and were born in England and Wales between January 1, 1994, and December 31, 1996 (details in eTable 1 in the Supplement and elsewhere^15^). The 11 108 twins included in the final sample (5894 girls and 5214 boys) were a mean age of 11.3 years at the first assessment and 16.3 years at the last assessment. The number of twins for each outcome ranged from 11 108 to 4706 (subsample at 14 years of age) depending on age, informant, and number of pairs with data available for childhood exposure to bullying and each outcome. Data collection took place when the participants were between 11 and 16 years of age from December 1, 2005, to January 31, 2013. Written informed consent was obtained from all participating families. This study was approved by the Institute of Psychiatry, Kings College London, Ethics Committee.

### Measures

Childhood exposure to bullying was measured using the self-report version of the Multidimensional Peer-Victimization Scale^16^ at 11 and 14 years of age. This 16-item measure comprises the following 4 subscales: physical bullying (eg, “Kicked me”), verbal bullying (eg, “Called me names”), social manipulation (eg, “Tried to make my friends turn against me”), and property attacks (eg, “Tried to break something of mine”). The twins rated how often they experienced events mentioned under each item during the past year on a 3-point scale (0 = not at all, 1 = once, and 2 = more than once). Cronbach α was 0.91 for the total scale and 0.80 to 0.84 for subscales.

Outcomes were measured at 11 and 16 years of age and included total mental health difficulties, anxiety, depression, hyperactivity and impulsivity, inattention, conduct problems, and psychotic-like experiences (ie, paranoid thoughts, hallucinations, grandiosity, cognitive disorganization, anhedonia, and negative symptoms). The questionnaires are described in Table 1,^17^ and Table 2 and Table 3 detail each outcome: timing of assessment, scale(s), and informant(s). eTables 2 and 3 in the Supplement contain findings from teacher ratings and outcomes that were excluded from main analyses (prosocial behavior and peer problems).

**Table 1.** Outcome Measures and Instruments

Outcome, Scale	Items, No.	Additional Information
Total difficulties		
SDQ^17^	15	Total difficulty score was derived from the Anxiety, Inattention-Hyperactivity, and Conduct Problems subscales of the SDQ. The Prosocial Behaviors subscale, which does not assess difficulties, was excluded. The Peer Problems subscale was also excluded to avoid content overlap between peer problem and exposure to bullying. Analyses for the Prosocial Behaviors and Peer Problems scales, as well as the total difficulty score including Peer Problems, are in eTables 2 and 3 in the Supplement.
Anxiety and depression		
Anxiety subscale (SDQ)	5	CASI and ARBQ assess anxiety, while MFQ assesses depressive symptoms.
CASI^18^	18	
ARBQ^19^	19	
MFQ^20^	11	
Inattention, hyperactivity and impulsivity		
Inattention-hyperactivity subscale of the SDQ	5	Conners scales are based on *DSM-IV* criteria. A total score was computed based on the 9 items for each dimension (18 items in total).
Inattention subscale of the Conners Parent Rating Scales–Revised^21^	9	
Hyperactivity-impulsivity (Conners)	9	
Conduct problems		
Conduct problems subscale (SDQ)	5	SDQ subscale for conduct problems.
Psychotic-like experiences		
Paranoid thoughts subscale of the SPEQ^22^	15	SPEQ was devised specifically to assess psychotic experiences in adolescence by adapting existing measures for adults, such as the Paranoia Checklist, to be suitable for adolescent participants.
Hallucinations (SPEQ)	9	
Grandiosity (SPEQ)	8	
Cognitive disorganization (SPEQ)	11	
Anhedonia (SPEQ)	10	
Negative symptoms (SPEQ)	10	

Abbreviations: ARBQ, Anxiety-Related Behaviors Questionnaire; CASI, Childhood Anxiety Sensitivity Index; *DSM-IV*, *Diagnostic and Statistical Manual of Mental Disorders* (Fourth Edition); MFQ, Moods and Feelings Questionnaire; SDQ, Strengths and Difficulties Questionnaire; SPEQ, Specific Psychotic Experiences Questionnaire.

**Table 2.** Contributions of Past-Year Exposure to Bullying at 11 Years of Age to Mental Health Outcomes at 11 Years of Age (ie, Concurrent Effect) and 16 Years of Age (ie, 5-Year Effect)

Outcome, Timing, Scale (Informant)	Total No. (DZSS, MZ)^a^	β (95% CI)		
		Phenotypic	DZ Differences	MZ Differences
Total difficulties				
Concurrent				
Total difficulties (SDQ-Parent)	5525 (1799, 2010)	0.233 (0.213 to 0.253)^b^	0.181 (0.130 to 0.232)^b^	0.043 (0.010 to 0.075)^b^
Total difficulties (SDQ-Child)	5522 (1799, 2012)	0.401 (0.382 to 0.420)^b^	0.348 (0.294 to 0.402)^b^	0.241 (0.189 to 0.294)^b^
5 y				
Total difficulties (SDQ-Child)	3807 (1241, 1403)	0.178 (0.154 to 0.203)^b^	0.143 (0.082 to 0.205)^b^	0.055 (−0.004 to 0.114)
Anxiety and depression				
Concurrent				
Anxiety (SDQ-Parent)	5525 (1798, 2010)	0.136 (0.116 to 0.157)^b^	0.124 (0.069 to 0.179)^b^	0.052 (0.002 to 0.101)^b^
Anxiety (SDQ-Child)	5521 (1798, 2012)	0.325 (0.304 to 0.345)^b^	0.308 (0.252 to 0.365)^b^	0.274 (0.216 to 0.332)^b^
Depression (MFQ-Parent)	5514 (1799, 2009)	0.193 (0.170 to 0.216)^b^	0.192 (0.135 to 0.253)^b^	0.096 (0.041 to 0.152)^b^
Depression (MFQ-Child)	5554 (1810, 2020)	0.427 (0.404 to 0.450)^b^	0.436 (0.373 to 0.499)^b^	0.377 (0.315 to 0.438)^b^
5 y				
Anxiety (ARBQ-Parent)	3818 (1245, 1407)	0.058 (0.034 to 0.084)^b^	0.052 (−0.004 to 0.113)	0.035 (−0.017 to 0.088)
Anxiety (SDQ-Child)	3854 (1249, 1421)	0.071 (0.047 to 0.096)^b^	0.083 (0.018 to 0.147)^b^	0.038 (−0.022 to 0.096)
Anxiety (CASI-Child)	3809 (1241, 1405)	0.097 (0.072 to 0.122)^b^	0.140 (0.077 to 0.206)^b^	0.023 (−0.038 to 0.080)
Depression (MFQ-Parent)	3851 (1249, 1418)	0.097 (0.072 to 0.124)^b^	0.065 (0.001 to 0.138)^b^	−0.023 (−0.095 to 0.031)
Depression (MFQ-Child)	3818 (1244, 1409)	0.124 (0.098 to 0.149)^b^	0.105 (0.034 to 0.174)^b^	0.034 (−0.035 to 0.105)
Inattention and hyperactivity-impulsivity				
Concurrent				
Hyperactivity (SDQ-Parent)	5525 (1799, 2010)	0.219 (0.198 to 0.239)^b^	0.149 (0.090 to 0.210)^b^	0.019 (−0.014 to 0.053)
Hyperactivity (SDQ-Child)	5522 (1799, 2012)	0.272 (0.253 to 0.292)^b^	0.218 (0.163 to 0.273)^b^	0.094 (0.042 to 0.147)^b^
Hyperactivity-impulsivity (Conners-Parent)	5531 (1804, 2007)	0.192 (0.170 to 0.214)^b^	0.155 (0.106 to 0.212)^b^	0.005 (−0.021 to 0.032)
Inattention (Conners-Parent)	5534 (1805, 2006)	0.228 (0.207 to 0.249)^b^	0.156 (0.103 to 0.214)^b^	0.037 (0.003 to 0.072)^b^
Total (Conners-Parent)	5533 (1805, 2007)	0.231 (0.210 to 0.253)^b^	0.173 (0.123 to 0.227)^b^	0.025 (−0.004 to 0.055)
5 y				
Hyperactivity (SDQ-Parent)	3842 (1246, 1417)	0.179 (0.152 to 0.204)^b^	0.131 (0.055 to 0.211)^b^	0.030 (−0.014 to 0.078)
Hyperactivity-impulsivity (Conners-Parent)	3849 (1247, 1420)	0.149 (0.121 to 0.178)^b^	0.131 (0.058 to 0.214)^b^	0.015 (−0.022 to 0.055)
Inattention (Conners-Parent)	3851 (1247, 1421)	0.184 (0.159 to 0.211)^b^	0.073 (0.000 to 0.147)^b^	0.043 (0.004 to 0.092)^b^
Total (Conners-Parent)	3851 (1247, 1421)	0.189 (0.163 to 0.216)^b^	0.110 (0.044 to 0.184)^b^	0.037 (0.000 to 0.079)^b^
Conduct problems				
Concurrent				
Conduct problems (SDQ-Parent)	5525 (1799, 2009)	0.184 (0.163 to 0.206)^b^	0.128 (0.076 to 0.182)^b^	0.027 (−0.006 to 0.063)
Conduct problems (SDQ-Child)	5523 (1799, 2012)	0.344 (0.323 to 0.364)^b^	0.282 (0.223 to 0.342)^b^	0.199 (0.140 to 0.259)^b^
5 y				
Conduct problems (SDQ-Parent)	3851 (1249, 1420)	0.134 (0.109 to 0.160)^b^	0.070 (0.003 to 0.144)^b^	0.002 (−0.047 to 0.052)
Conduct problems (SDQ-Child)	3807 (1241, 1404)	0.174 (0.149 to 0.200)^b^	0.116 (0.038 to 0.189)^b^	0.018 (−0.051 to 0.087)
Psychotic-like experiences				
5 y				
Paranoid thoughts (SPEQ-Child)	3813 (1243, 1404)	0.209 (0.182 to 0.235)^b^	0.152 (0.086 to 0.217)^b^	0.075 (0.016 to 0.136)^b^
Hallucinations (SPEQ-Child)	3817 (1245, 1408)	0.146 (0.120 to 0.171)^b^	0.080 (0.007 to 0.150)^b^	0.059 (−0.009 to 0.128)
Grandiosity (SPEQ-Child)	3813 (1242, 1406)	0.044 (0.019 to 0.068)^b^	0.009 (−0.056 to 0.075)	0.005 (−0.057 to 0.069)
Cognitive disorganization (SPEQ-Child)	3806 (1238, 1405)	0.139 (0.115 to 0.163)^b^	0.124 (0.059 to 0.189)^b^	0.091 (0.031 to 0.150)^b^
Anhedonia (SPEQ-Child)	3807 (1238, 1405)	0.111 (0.087 to 0.134)^b^	0.033 (−0.033 to 0.097)	0.017 (−0.054 to 0.085)
Negative symptoms (SPEQ-Parent)	3849 (1247, 1419)	0.096 (0.071 to 0.122)^b^	0.002 (−0.066 to 0.073)	0.023 (−0.013 to 0.058)

Abbreviations: ARBQ, Anxiety-Related Behaviors Questionnaire: CASI, Childhood Anxiety Sensitivity Index; DZ, dizygotic; DZSS, DZ same-sex twins; MFQ, Moods and Feelings Questionnaire; MZ, monozygotic; SDQ, Strengths and Difficulties Questionnaire; SPEQ, Specific Psychotic Experiences Questionnaire.

^a^ Opposite-sex twin pairs were excluded from the DZ analyses to control for sex.

^b^ Significant estimate.

**Table 3.** Contribution of Exposure to Bullying at 14 Years of Age to Mental Health at 16 Years of Age (ie, 2-Year Effect)

Outcome, Scale (Informant)	Total No. (DZSS, MZ)^a^	β (95% CI)		
		Phenotypic	DZ Differences	MZ Differences
Total difficulties				
Total difficulties (SDQ-Child)	2353 (759, 929)	0.238 (0.205 to 0.271)^b^	0.238 (0.154 to 0.327)^b^	0.106 (0.021 to 0.187)^b^
Anxiety (ARBQ-Parent)	2387 (767, 940)	0.078 (0.047 to 0.112)^b^	0.051 (−0.008 to 0.112)	0.079 (0.015 to 0.159)^b^
Anxiety (SDQ-Child)	2354 (759, 930)	0.129 (0.097 to 0.161)^b^	0.117 (0.034 to 0.193)^b^	0.117 (0.042 to 0.195)^b^
Anxiety (CASI-Child)	2364 (766, 930)	0.131 (0.099 to 0.164)^b^	0.132 (0.058 to 0.214)^b^	0.146 (0.065 to 0.220)^b^
Depression (MFQ-Parent)	2385 (767, 937)	0.125 (0.092 to 0.161)^b^	0.101 (0.041 to 0.172)^b^	0.028 (−0.060 to 0.113)
Depression (MFQ-Child)	2363 (764, 930)	0.189 (0.156 to 0.223)^b^	0.163 (0.094 to 0.239)^b^	0.069 (−0.033 to 0.161)
Inattention and hyperactivity-impulsivity				
Hyperactivity (SDQ-Parent)	2378 (765, 937)	0.173 (0.141 to 0.205)^b^	0.112 (0.034 to 0.192)^b^	0.035 (−0.025 to 0.107)
Hyperactivity-impulsivity (Conners-Parent)	2381 (765, 937)	0.134 (0.100 to 0.172)^b^	0.067 (0.006 to 0.134)^b^	−0.002 (−0.052 to 0.072)
Inattention (Conners-Parent)	2382 (765, 938)	0.185 (0.152 to 0.219)^b^	0.099 (0.031 to 0.169)^b^	0.041 (−0.011 to 0.118)
Total (Conners-Parent)	2382 (765, 938)	0.185 (0.151 to 0.221)^b^	0.097 (0.034 to 0.164)^b^	0.027 (−0.022 to 0.114)
Conduct problems				
Conduct problems (SDQ-Parent)	2384 (767, 939)	0.155 (0.121 to 0.188)^b^	0.123 (0.054 to 0.198)^b^	0.033 (−0.020 to 0.087)
Conduct problems (SDQ-Child)	2353 (759, 930)	0.222 (0.190 to 0.255)^b^	0.210 (0.108 to 0.314)^b^	0.056 (−0.044 to 0.152)
Psychotic-like experiences				
Paranoid thoughts (SPEQ-Child)	2362 (765, 928)	0.342 (0.308 to 0.377)^b^	0.252 (0.179 to 0.327)^b^	0.241 (0.158 to 0.333)^b^
Hallucinations (SPEQ-Child)	2363 (765, 930)	0.213 (0.179 to 0.247)^b^	0.149 (0.073 to 0.225)^b^	0.119 (0.028 to 0.214)^b^
Grandiosity (SPEQ-Child)	2360 (765, 928)	0.057 (0.026 to 0.088)^b^	0.075 (−0.014 to 0.159)	−0.077 (−0.155 to 0.001)
Cognitive disorganization (SPEQ-Child)	2360 (762, 930)	0.194 (0.165 to 0.225)^b^	0.215 (0.142 to 0.288)^b^	0.146 (0.064 to 0.226)^b^
Anhedonia (SPEQ-Child)	2359 (762, 929)	0.152 (0.121 to 0.183)^b^	0.069 (−0.016 to 0.151)	0.047 (−0.043 to 0.135)
Negative symptoms (SPEQ-Parent)	2383 (767, 936)	0.094 (0.062 to 0.127)^b^	−0.011 (−0.074 to 0.047)	0.009 (−0.040 to 0.060)

Abbreviations: ARBQ, Anxiety-Related Behaviors Questionnaire: CASI, Childhood Anxiety Sensitivity Index; DZ, dizygotic; DZSS, DZ same-sex twins; MFQ, Moods and Feelings Questionnaire; MZ, monozygotic; SDQ, Strengths and Difficulties Questionnaire; SPEQ, Specific Psychotic Experiences Questionnaire.

^a^ Opposite-sex twin pairs were excluded from the DZ analyses to control for sex.

^b^ Significant estimate.

### Statistical Analyses

Statistical analysis was conducted from January 1, 2016, to June 20, 2017. Three main types of estimates of the relationship between childhood exposure to bullying and each outcome were obtained: unadjusted phenotypic estimate, estimate from twin differences in DZ same-sex twins, and estimate from twin differences in MZ twins.^23^

For phenotypic estimates on the entire sample, the nonindependence within twin pairs was accounted for by allowing for a within-twin correlation.^23^ Maximum likelihood estimates were obtained in the Structural Equation Modeling Lavaan package, version 0.5-20, in R.^25^ For DZ and MZ estimates, an ordinary least square through origin regression (ie, without the intercept) was conducted, regressing within-twin differences in outcomes on within-twin differences in childhood exposure to bullying.^23^ Positive regression estimates mean that the twin who was more exposed to bullying also presented with higher levels of mental health difficulties. To account for nonnormality and nonindependence, robust 95% CIs were obtained by bootstrapping (10 000 repetitions).

Dizygotic twins share 50% of their segregated genes on average and 100% of shared environmental influences. Similar to a fixed-effect sibling design, DZ estimates are therefore more stringent than phenotypic estimates because they account partly for genetic confounding (eg, prior genetically influenced individual vulnerabilities) and account completely for shared environmental influences. Monozygotic twins share 100% of their genes and shared environmental influences; therefore, MZ estimates represent a further improvement compared with DZ estimates. Although they are extremely stringent, MZ analyses do not account for within-twin differences that can arise from nonshared environmental factors preceding exposure to bullying. For example, a preexisting vulnerability for anxiety caused by nonshared environmental factors in one twin compared to his or her co-twin may at the same time evoke exposure to higher levels of bullying and explain later levels of anxiety. For each analysis, we therefore conducted an additional analysis controlling for within-twin differences at earlier ages (eg, controlling for prior within-twin differences in anxiety when examining the direct contribution of exposure to bullying to anxiety outcomes). When the corresponding measure was unavailable (eg, for paranoid thoughts), we used the total difficulty score instead. We considered parenting variables as additional potential sources of nonshared environmental confounding; parenting appeared unlikely to bias MZ estimates (eTable 4 in the Supplement).

### Longitudinal Analyses

We conducted 3 sets of analyses: concurrent, 2 years after exposure to bullying, and 5 years after exposure to bullying. In concurrent analyses, both the childhood exposure to bullying and the outcomes were measured at 11 years of age. In the 2-year analyses, we used the subset of participants for whom exposure to bullying was assessed at age 14 years and outcomes at age 16 years. In the 5-year analyses, exposure to bullying was measured at 11 years of age and outcomes at 16 years of age. Analyses were conducted with the overall exposure to bullying score and repeated for each subscale. We further probed the relationships between exposure to bullying and mental health by testing for moderation by sex and nonlinear associations.

## Results

The Figure provides an illustration of the 3 types of estimates—phenotypic, DZ, and MZ—for the concurrent, 2-year, and 5-year analyses. Based on the total mental health difficulties score, the Figure also illustrates key patterns of the study findings. First, the decrease in effect sizes from phenotypic to DZ and MZ estimates shows the importance of shared environmental and/or genetic confounding in explaining observed associations. Despite this decrease, MZ estimates remained significant for the concurrent and 2-year analyses, supporting an association between childhood exposure to bullying and total difficulties. Second, this direct contribution of childhood exposure to bullying decreased as time from the exposure increased. Findings for all outcomes are presented below in chronological order.

**Figure.**
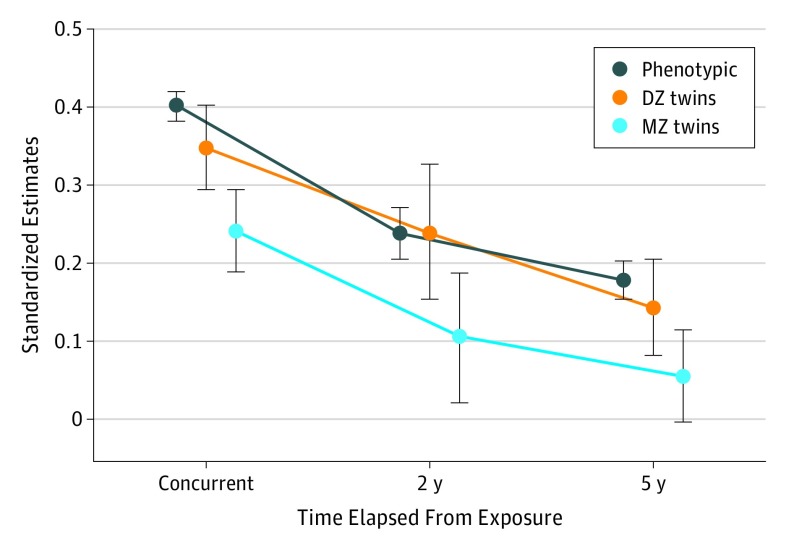
Longitudinal Contribution of Exposure to Bullying in Childhood to Child-Rated Total Mental Health Difficulties The decrease in size from phenotypic estimates to the most stringent monozygotic (MZ) estimates (eg, 3 concurrent estimates) and the decrease of estimates as time from the exposure increases are displayed. Childhood exposure to bullying and mental health outcomes were assessed at the following ages: 11 years (concurrent), 14 and 16 years (2 years), and 11 and 16 years (5 years). The whiskers above and below each estimate indicate the 95% CI. DZ indicates dizygotic.

### Concurrent Estimates

Table 2 presents phenotypic, DZ, and MZ concurrent estimates at 11 years of age arranged by outcome, age, scale, and informant. Findings from teacher ratings (eTable 2 in the Supplement) were largely consistent with parent ratings. Descriptives are presented in eTable 5 in the Supplement. Phenotypic estimates showed that childhood exposure to bullying in the past year was significantly associated with all mental health outcomes across all informants. Most of these relationships remained significant when controlling for all shared environmental influences and half of the genetic influences in DZ analyses. Monozygotic estimates were consistent with a causal influence of childhood exposure to bullying on the total difficulty score, depression, and anxiety across all informants. In addition, MZ estimates were also significant for child-rated conduct problems, child-rated hyperactivity and inattention symptoms as measured by the Strengths and Difficulties Questionnaire,^17^ and parent-rated inattention (but not hyperactivity and impulsivity) from the Conners scale.^21^ Findings were similar when further controlling for prior within-twin differences (eTable 6 in the Supplement).

### Two-Year Estimates (Subsample Analysis)

Findings and descriptives for 2-year estimates (from exposure to bullying at 14 years of age to outcomes at 16 years of age) are in Table 3 and eTables 3 and 7 in the Supplement. All phenotypic estimates remained significant. Effect sizes tended to lie between the concurrent and 5-year estimates (Figure). In MZ analyses, the total difficulty score, child-rated and parent-rated anxiety on 3 different scales (Strengths and Difficulties Questionnaire, Childhood Anxiety Sensitivity Index, and Anxiety-Related Behaviors Questionnaire), paranoid thoughts, hallucinations, and cognitive disorganization were significant. When further controlling for prior within-twin differences (eTable 8 in the Supplement), child-rated and parent-rated anxiety, paranoid thoughts, and cognitive disorganization remained significant (for paranoid thoughts and cognitive disorganization, there were no prior corresponding measures, so the total difficulty score was used instead).

### Five-Year Estimates

Findings and descriptives for 5-year estimates (from exposure to bullying at 11 years of age to outcomes at 16 years of age) are in Table 2 and eTables 2 and 9 in the Supplement. All phenotypic estimates remained significant, although they were smaller than concurrent and 2-year estimates. However, in the MZ analyses, only paranoid thoughts, cognitive disorganization, and the total score and inattention score on the Conners scale were still significant. The total score and inattention score on the Conners scale did not survive additional control for early within-twin differences in these behaviors (eTable 10 in the Supplement).

### Additional Analyses

Analyses for the physical subscale are in eTables 11 to 13 in the Supplement, analyses for the verbal subscale are in eTables 14 to 16 in the Supplement, analyses for the social subscale are in eTables 17 to 19 in the Supplement, and analyses for the property-related subscale are in eTables 20 to 22 in the Supplement. Intercorrelations between subscales are in eTables 23 and 24 in the Supplement. Overall, findings for the 4 subscales were consistent with findings for the total exposure to bullying score in terms of significance and timing of direct contributions, particularly for physical and social bullying. We found no robust evidence of moderation by sex or nonlinear relationships (eTables 25-27 in the Supplement).

## Discussion

We have provided stringent evidence that childhood exposure to bullying directly contributes to multiple mental health domains. In particular, findings were consistent across multiple informants and multiple scales for concurrent depression and anxiety. Increased levels of anxiety persisted in the short term (2 years), while findings indicated a small but enduring contribution of exposure to bullying in childhood to paranoid thoughts and cognitive disorganization.

### Exposure to Bullying and Mental Health: Confounding and Causation

In line with extant research, we found widespread phenotypic associations between childhood exposure to bullying and mental health, with all estimates being significant. Most estimates were reduced but remained significant in analyses of DZ twin differences. However, few estimates survived the most stringent MZ analyses, which control entirely for shared environmental and genetic influences, particularly when further controlling for preexisting individual mental health vulnerabilities. Overall, this pattern of findings suggests that reported associations between childhood exposure to bullying and mental health outcomes likely reflect, at least in part, multiple vulnerabilities of bullied individuals rather than a causal contribution of childhood exposure to bullying. Furthermore, all phenotypic estimates but very few MZ estimates remained significant in the 5-year period. Causal contributions may therefore be shorter lived than confounded associations. These findings underscore recent calls for the use of more stringent causal inference designs in developmental psychiatry,^26^ particularly when assessing the long-term consequences of childhood exposure to bullying.^9^

The MZ twin differences design provided strong evidence of the concurrent contribution of exposure to bullying in childhood to the total difficulty score, depression, and anxiety. Findings were consistent across informants and scales. The 2 previous discordant MZ twin studies reported significant contributions to overall internalizing problems^13^ as well as social and separation anxiety^14^ in childhood. We also found evidence of a concurrent contribution of exposure to bullying in childhood to hyperactivity and inattention symptoms, as well as conduct problems. However, these contributions were not consistent across scales and informants and should be interpreted with caution. One previous MZ discordant twin study also found a concurrent contribution to attention-deficit/hyperactivity disorder but not to conduct disorder, and did not examine long-term contributions for these 2 outcomes.^14^ Taken together, these findings represent the most stringent evidence to date, to our knowledge, of the immediate detrimental contribution of exposure to bullying to children’s mental health. In addition, beyond the documented genetic correlation between childhood exposure to bullying and paranoid thoughts,^30^ our twin differences analyses suggest that exposure to bullying in childhood affects paranoid thoughts and cognitive disorganization in adolescence, although this may not persist into adulthood.^31^

The present findings can guide targeted research aiming to uncover mechanisms underlying the contribution of exposure to bullying in childhood to anxiety, paranoid thoughts, and cognitive disorganization. Promising candidate mechanisms can be investigated at multiple levels: altered neurocognitive profiles in children who experience bullying (eg, modification in threat and trust processing leading to paranoid thinking), alterations in brain response (eg, stress axis), or epigenetic mechanisms.^32^

### Childhood Exposure to Bullying and Resilience

As time elapsed from exposure, the direct contributions of exposure to bullying in childhood to mental health dissipated. Most contributions were not maintained after 2 years. Particularly striking were the strong concurrent contributions to anxiety that were reduced but still present across informants after 2 years, which had dissipated entirely after 5 years. Similarly, direct contributions to paranoid thoughts and cognitive disorganization were smaller for the 5-year vs the 2-year period. This pattern of findings highlights the potential for resilience in children exposed to bullying. Consequently, a more hopeful message can be delivered to children and families, acknowledging the suffering endured by children being bullied, while supporting resilience processes on their path to recovery. Further studies should seek to establish fine-grained timing toward resilience. Kelleher et al^34^ reported a decrease in psychotic-like experiences as rapidly as 3 months after the bullying had ceased. Furthermore, future studies should aim to identify protective modifiable factors, such as school support, that may facilitate rapid recovery.^35^

### Implications for Interventions

Interventions designed to prevent exposure to bullying remain important to avoid prolonged exposure to an experience that can induce anxiety and depression. However, such interventions have not proven to be universally effective in reducing the level of exposure to bullying,^36^ and complementary approaches are required to best help children and young people. Our findings highlight the importance of preexisting vulnerabilities (eg, previous mental health difficulties), which in part account for the associations between childhood exposure to bullying and mental health. Exposure to bullying may be viewed not only as a cause of adverse mental health but may also in part represent a “symptom” of preexisting vulnerabilities. This finding has implications for secondary prevention of mental health difficulties in children exposed to bullying. Specifically, we must be mindful in any prevention effort that our goal should be not only to stop the bullying but also to address potential preexisting vulnerabilities to improve mental health in the long term. We propose that combining programs of childhood bullying prevention as well as individual work with vulnerable children by addressing existing mental health problems and promoting resilience will yield the best outcomes. Such work must be undertaken sensitively to ensure that children exposed to childhood bullying are not in any way seen as responsible for being bullied. Rather, these findings simply indicate what is commonly understood in clinical and educational settings: that some children are more vulnerable and require greater support to meet their full potential.

### Limitations

This study has some limitations. Although it is considerably more stringent than nongenetically informative observational designs, the twin differences design does not account for nonshared environmental confounding factors, which might exaggerate the contribution of childhood exposure to bullying. To reduce this bias, we adjusted for prior within-pair differences in mental health difficulties. However, such prior measures were not available for all outcomes, particularly for paranoid thoughts and cognitive disorganization. It is therefore possible that preexisting paranoid tendencies owing to nonshared environmental factors affected the reporting of exposure to bullying. Although we carefully considered the possibility of nonshared environmental confounding, we were unable to control adequately for other forms of bullying (eg, sexual bullying), which might have overestimated the independent role of childhood exposure to bullying. In addition, multiple-informant, multiscale assessments were not available for all outcomes. Therefore, we could not account for shared method variance bias equally well for all outcomes. Despite modest differences in demographic characteristics between the samples used in the analyses, the level of attrition may have influenced the findings. Finally, our findings do not entirely preclude the existence of long-term causal relationships, as childhood exposure to bullying may contribute to unmeasured mental health outcomes^10^ and outcomes outside mental health,^29^ and contributions may be limited to subpopulations.

## Conclusions

We reported robust evidence of the direct contribution of exposure to bullying in childhood to symptoms of depression and anxiety, as well as indications of a contribution to paranoid thoughts and cognitive disorganization. Our finding that this direct contribution dissipated or reduced over time highlights the potential for resilience in children exposed to bullying. This finding also highlights the need for further investigations into mechanisms of resilience that could be harnessed in future interventions. In addition to primary prevention aiming to stop exposure to bullying, secondary preventive interventions in children exposed to bullying should address prior vulnerabilities, such as mental health difficulties, if we are to achieve a long-term impact on mental health.

## Notes

Supplement. **eTable 1.** Comparison of Current Study With TEDS Initial Sample and United Kingdom Census Characteristics **eTable 2.** Extended Findings for Phenotypic Analyses, DZ (Same-Sex), MZ Twin Differences Analyses for Peer Victimization at Concurrent, and Mental Health Outcomes at Concurrent and 16 (ie, Concurrent and Five-Year Analyses) **eTable 3.** Results From the Phenotypic Analyses, DZ (Same-Sex), and MZ Twin Differences Analyses for Victimization at Age 14 and Mental Health Outcomes at Age 16 (ie, Two-Year Analyses) **eTable 4.** Consideration of Early Parenting Variables **eTable 5.** Descriptives for Outcome Measures for Concurrent Analyses (Victimization and Outcomes Assessed at 11 Years) **eTable 6.** Concurrent MZ Analyses Repeated With Further Control for Preexisting Twin Differences in Mental Health Difficulties at Ages 7 and 9 Years **eTable 7.** Descriptives Two-Year Analyses (Victimization Assessed at Age 14 Years and Outcomes at 16 Years) **eTable 8.** Two-Year MZ Analyses Repeated With Further Control for Preexisting Twin Differences in Mental Health Difficulties at Ages 7 and 9 Years **eTable 9.** Descriptives for Five-Year Analyses (Victimization Assessed at Age 11 Years and Outcomes at 16 Years) **eTable 10.** Five-Year MZ Analyses Repeated With Further Control for Preexisting Twin Differences in Mental Health Difficulties at Ages 7 and 9 Years **eTable 11.** Concurrent Analyses With Physical Victimization **eTable 12.** Five-Year Analyses With Physical Victimization **eTable 13.** Two-Year Analyses With Physical Victimization **eTable 14.** Concurrent Analyses With Verbal Victimization **eTable 15.** Five-Year Analyses With Verbal Victimization **eTable 16.** Two-Year Analyses With Verbal Victimization **eTable 17.** Concurrent Analyses With Social Victimization **eTable 18.** Five-Year Analyses With Social Victimization **eTable 19.** Two-Year Analyses With Social Victimization **eTable 20.** Concurrent Analyses With Property-Related Victimization **eTable 21.** Five-Year Analyses With Property-Related Victimization **eTable 22.** Two-Year Analyses With Property-Related Victimization **eTable 23.** Intercorrelations of Victimization Subscales at Concurrent **eTable 24.** Intercorrelations of Victimization Subscales at Age 14 Years **eTable 25.** Moderation by Sex of Phenotypic Estimates, for Peer Victimization at Age 11 Years and Mental Health Outcomes at Age 11 Years (ie, Concurrent Effect) and at Age 16 Years (ie, Five-Year Effect) **eTable 26.** Moderation by Sex of Phenotypic Estimates: Same-Sex Twin Differences for Peer Victimization at Age 14 Years and Mental Health Outcomes at Age 16 Years (ie, Two-Year Analyses) **eTable 27.** Linear and Quadratic Effects in Phenotypic Analyses, Peer Victimization at Age 11 Years and Mental Health Outcomes at Age 11 Years (ie, Concurrent Effect) and at Age 16 Years (ie, Five-Year Effect)
eReferences.
