# Supplementary material for: Concurrent and Longitudinal Contribution of Exposure to Bullying in Childhood to Mental Health: The Role of Vulnerability and Resilience
Source: JAMA Psychiatry. 2017 Nov 1;74(11):1112–9. doi: 10.1001/jamapsychiatry.2017.2678 (PMC5710218; doi:10.1001/jamapsychiatry.2017.2678)
Supplement: Supplement. — eTable 1. Comparison of Current Study With TEDS Initial Sample and United Kingdom Census Characteristics eTable 2. Extended Findings for Phenotypic Analyses, DZ (Same-Sex), MZ Twin Differences Analyses for Peer Victimization at Concurrent, and Mental Health Outcomes at Concurrent and 16 (ie, Concurrent and Five-Year Analyses) eTable 3. Results From the Phenotypic Analyses, DZ (Same-Sex), and MZ Twin Differences Analyses for Victimization at Age 14 and Mental Health Outcomes at Age 16 (ie, Two-Year Analyses) eTable 4. Consideration of Early Parenting Variables eTable 5. Descriptives for Outcome Measures for Concurrent Analyses (Victimization and Outcomes Assessed at 11 Years) eTable 6. Concurrent MZ Analyses Repeated With Further Control for Preexisting Twin Differences in Mental Health Difficulties at Ages 7 and 9 Years eTable 7. Descriptives Two-Year Analyses (Victimization Assessed at Age 14 Years and Outcomes at 16 Years) eTable 8. Two-Year MZ Analyses Repeated With Further Control for Preexisting Twin Differences in Mental Health Difficulties at Ages 7 and 9 Years eTable 9. Descriptives for Five-Year Analyses (Victimization Assessed at Age 11 Years and Outcomes at 16 Years) eTable 10. Five-Year MZ Analyses Repeated With Further Control for Preexisting Twin Differences in Mental Health Difficulties at Ages 7 and 9 Years eTable 11. Concurrent Analyses With Physical Victimization eTable 12. Five-Year Analyses With Physical Victimization eTable 13. Two-Year Analyses With Physical Victimization eTable 14. Concurrent Analyses With Verbal Victimization eTable 15. Five-Year Analyses With Verbal Victimization eTable 16. Two-Year Analyses With Verbal Victimization eTable 17. Concurrent Analyses With Social Victimization eTable 18. Five-Year Analyses With Social Victimization eTable 19. Two-Year Analyses With Social Victimization eTable 20. Concurrent Analyses With Property-Related Victimization eTable 21. Five-Year Analyses With Property-Related Victimization eTable 22. T [file jamapsychiatry-74-1112-s001.pdf]

Supplementary Online Content

Singham T, Viding E, Schoeler T, et al. Concurrent and longitudinal contribution of exposure to bullying in childhood to mental health: the role of vulnerability and resilience. *JAMA Psychiatry.* Published online October 4, 2017. doi:10.1001/jamapsychiatry.2017.2678

eTable 1. Comparison of Current Study With TEDS Initial Sample and United Kingdom Census Characteristics
eTable 2. Extended Findings for Phenotypic Analyses, DZ (Same-Sex), MZ Twin Differences Analyses for Peer Victimization at Concurrent, and Mental Health Outcomes at Concurrent and 16 (i.e. Concurrent and Five-Year Analyses)
eTable 3. Results From the Phenotypic Analyses, DZ (Same-Sex), and MZ Twin Differences Analyses for Victimization at Age 14 and Mental Health Outcomes at Age 16 (i.e. Two-Year Analyses)
eTable 4. Consideration of Early Parenting Variables
eTable 5. Descriptives for Outcome Measures for Concurrent Analyses (Victimization and Outcomes Assessed at 11 Years)
eTable 6. Concurrent MZ Analyses Repeated With Further Control for Preexisting Twin Differences in Mental Health Difficulties at Ages 7 and 9 Years
eTable 7. Descriptives Two-Year Analyses (Victimization Assessed at Age 14 Years and Outcomes at 16 Years)
eTable 8. Two-Year MZ Analyses Repeated With Further Control for Preexisting Twin Differences in Mental Health Difficulties at Ages 7 and 9 Years
eTable 9. Descriptives for Five-Year Analyses (Victimization Assessed at Age 11 Years and Outcomes at 16 Years)
eTable 10. Five-Year MZ Analyses Repeated With Further Control for Preexisting Twin Differences in Mental Health Difficulties at Ages 7 and 9 Years
eTable 11. Concurrent Analyses With Physical Victimization
eTable 12. Five-Year Analyses With Physical Victimization
eTable 13. Two-Year Analyses With Physical Victimization
eTable 14. Concurrent Analyses With Verbal Victimization
eTable 15. Five-Year Analyses With Verbal Victimization
eTable 16. Two-Year Analyses With Verbal Victimization
eTable 17. Concurrent Analyses With Social Victimization
eTable 18. Five-Year Analyses With Social Victimization
eTable 19. Two-Year Analyses With Social Victimization
eTable 20. Concurrent Analyses With Property-Related Victimization
eTable 21. Five-Year Analyses With Property-Related Victimization
eTable 22. Two-Year Analyses With Property-Related Victimization
eTable 23. Intercorrelations of Victimization Subscales at Concurrent
eTable 24. Intercorrelations of Victimization Subscales at Age 14 Years
eTable 25. Moderation by Sex of Phenotypic Estimates, for Peer Victimization at Age 11 Years and Mental Health Outcomes at Age 11 Years (i.e. Concurrent Effect) and at Age 16 Years (i.e. Five-Year Effect)
eTable 26. Moderation by Sex of Phenotypic Estimates: Same-Sex Twin Differences for Peer Victimization at Age 14 Years and Mental Health Outcomes at Age 16 Years (i.e. Two-Year Analyses)
eTable 27. Linear and Quadratic Effects in Phenotypic Analyses, Peer Victimization at Age 11 Years and Mental Health Outcomes at Age 11 Years (i.e. Concurrent Effect) and at Age 16 Years (i.e. Five-Year Effect)
eReferences.

© 2017 Singham T et al. *JAMA Psychiatry.*

This supplementary material has been provided by the authors to give readers additional information about their work.

© 2017 Singham T et al. *JAMA Psychiatry*.

eTable 1. Comparison of Current Study With TEDS Initial Sample and United Kingdom Census Characteristics

	Returned data (N families)	% white	% Mothers with A-levels or higher	% Mother employed	% Father employed	% Female	% MZ
UK Census	-	93%	32%	49%	89%	-	-
TEDS first contact	12593	92.90%	35.50%	43.10%	91.60%	50.10%	33.10%
Current study sample	5554	93.40%	43.70%	47.30%	93.50%	53.10%	36.40%
MFQ-Depression (Parent) 11-11yr	5514	93.60%	43.70%	47.20%	93.60%	53.10%	36.40%
SPEQ-Cognitive Disorganisation (Child) 11-16yr	3806	94.10%	47.30%	47.40%	94.20%	55.80%	36.90%
Total Difficulties (Child) 14-16yr	2353	94.60%	47.60%	47.20%	94.80%	56.30%	39.50%

Note. The Twins Early Development Study (TEDS). Twin births between 1994 and 1996 were identified through birth records by the United Kingdom Office for National Statistics. A total of 16,810 families responded to the office to acknowledge their interest in taking part in the TEDS study. A first contact with these families was made by TEDS team when the twins were about 18 months. A total of 12,593 families returned data for this first contact. eTable1 (above) shows the characteristics of these respondents. These characteristics mirror very closely the data from the U.K. census data. UK Census data from the 2000 General Household Survey is used rather than more recent data because they provide more appropriate comparisons for the TEDS Twins used in this sample who were born between 1994-1996. As such, TEDS families appear reasonably representative of the UK population. **For the present study,** twins who did not provide consent on first contact with TEDS, with unknown zygosity, or with severe medical or neurological problems were excluded from all analyses. The characteristics of the 5,554 families included in the current study sample are presented also in eTable 1 (above) and match closely with the first contact characteristics. The biggest difference is maternal education, with an 8.20 percentage point difference between mothers with A-levels of education or higher in the study sample as compared to the TEDS first contact sample. MZ = Monozygotic Twins. The three last rows present the characteristics for three subsamples, that is for the outcomes with the lowest N for our three longitudinal analyses (concurrent, two-year and five-year).

© 2017 Singham T et al. *JAMA Psychiatry*.

eTable 2. Extended Findings for Phenotypic Analyses, DZ (Same-Sex), MZ Twin Differences Analyses for Peer Victimization at Concurrent, and Mental Health Outcomes at Concurrent and 16 (i.e. Concurrent and Five-Year Analyses)

Outcome	Timing	Variable Name	Total N (DZSS, MZ)	Phenotypic		DZ Differences		MZ Differences	
				B	95% CI	B	95% CI	B	95% CI
Total Difficulties	Concurrent	SDQ Total Difficulties (Parent)	5525 (1799,2010)	**.233**	**(.213,.253)**	**.181**	**(.130,.232)**	**.043**	**(.010,.075)**
		SDQ Total Difficulties (Teacher)	3549 (1153,1313)	**.207**	**(.180,.235)**	**.108**	**(.038,.182)**	**.088**	**(.030,.144)**
		SDQ Total Difficulties (Child)	5522 (1799,2012)	**.401**	**(.382,.420)**	**.348**	**(.294,.402)**	**.241**	**(.189,.294)**
	Five-year	SDQ Total Difficulties (Child)	3807 (1241,1403)	**.178**	**(.154,.203)**	**.143**	**(.082,.205)**	.055	(-.004,.114)
Total Difficulties (Including peer problems)	Concurrent	SDQ including Peer Problems (Parent)	5526 (1799,2010)	**.270**	**(.250,.292)**	**.228**	**(.180,.277)**	**.066**	**(.035,.098)**
		SDQ including Peer Problems (Teacher)	3560 (1156,1315)	**.224**	**(.196,.253)**	**.130**	**(.066,.198)**	**.122**	**(.069,.178)**
		SDQ including Peer Problems (Child)	5523 (1799,2012)	**.457**	**(.438,.476)**	**.414**	**(.362,.468)**	**.276**	**(.222,.329)**
	Five-year	SDQ including Peer Problems (Child)	3808 (1241,1404)	**.201**	**(.178,.226)**	**.155**	**(.094,.217)**	**.065**	**(.006,.122)**
Anxiety & Depression	Concurrent	SDQ-Anxiety (Parent)	5525 (1798,2010)	**.136**	**(.116,.157)**	**.124**	**(.069,.179)**	**.052**	**(.002,.101)**
		SDQ-Anxiety (Teacher)	3553 (1152,1316)	**.147**	**(.120,.174)**	**.122**	**(.050,.195)**	**.146**	**(.079,.217)**
		SDQ-Anxiety (Child)	5521 (1798,2012)	**.325**	**(.304,.345)**	**.308**	**(.252,.365)**	**.274**	**(.216,.332)**
		MFQ-Depression (Parent)	5514 (1799,2009)	**.193**	**(.170,.216)**	**.192**	**(.135,.253)**	**.096**	**(.041, .152)**
		MFQ-Depression (Child)	5554 (1810,2020)	**.427**	**(.404,.450)**	**.436**	**(.373,.499)**	**.377**	**(.315,.438)**
	Five-year	SDQ-Anxiety (Child)	3854 (1249,1421)	**.071**	**(.047,.096)**	**.083**	**(.018,.147)**	.038	(-.022,.096)
		ARBQ-Anxiety (Parent)	3818 (1245,1407)	**.058**	**(.034,.084)**	.052	(-.004,.113)	.035	(-.017,.088)
		CASI-Anxiety (Child)	3809 (1241,1405)	**.097**	**(.072,.122)**	**.140**	**(.077,.206)**	.023	(-.038,.080)
		MFQ-Depression (Parent)	3851 (1249,1418)	**.097**	**(.072,.124)**	**.065**	**(.001,.138)**	-.023	(-.095,.031)
		MFQ-Depression (Child)	3818 (1244,1409)	**.124**	**(.098,.149)**	**.105**	**(.034,.174)**	.034	(-.035,.105)
Hyper-activity / Inattention / Impulsiveness	Concurrent	SDQ-Hyperactivity (Parent)	5525 (1799,2010)	**.219**	**(.198,.239)**	**.149**	**(.090,.210)**	.019	(-.014,.053)
		SDQ-Hyperactivity (Teacher)	3560 (1156,1316)	**.171**	**(.145,.197)**	**.081**	**(.014,.150)**	.037	(-.017,.093)
		SDQ-Hyperactivity (Child)	5522 (1799,2012)	**.272**	**(.253,.292)**	**.218**	**(.163,.273)**	**.094**	**(.042,.147)**
		Conners Total (Parent)	5533 (1805,2007)	**.231**	**(.210,.253)**	**.173**	**(.123,.227)**	.025	(-.004,.055)
		Conners Hyp-Impul (Parent)	5531 (1804,2007)	**.192**	**(.170,.214)**	**.155**	**(.106,.212)**	.005	(-.021,.032)
		Conners Inatt (Parent)	5534 (1805,2006)	**.228**	**(.207,.249)**	**.156**	**(.103,.214)**	**.037**	**(.003,.072)**
	Five-year	SDQ-Hyperactivity (Parent)	3842 (1246,1417)	**.179**	**(.152,.204)**	**.131**	**(.055,.211)**	.030	(-.014,.078)
		SDQ-Hyperactivity (Child)	3807 (1241,1403)	**.173**	**(.150,.196)**	**.120**	**(.052,.188)**	.055	(-.012,.121)
		Conners Total (Parent)	3851 (1247,1421)	**.189**	**(.163,.216)**	**.110**	**(.044,.184)**	**.037**	**(.000,.079)**
		Conners Hyp-Impul (Parent)	3849 (1247,1420)	**.149**	**(.121,.178)**	**.131**	**(.058,.214)**	.015	(-.022,.055)
		Conners Inatt (Parent)	3851 (1247,1421)	**.184**	**(.159,.211)**	**.073**	**(.000,.147)**	**.043**	**(.004,.092)**
Conduct Problems	Concurrent	SDQ-CP (Parent)	5525 (1799,2009)	**.184**	**(.163,.206)**	**.128**	**(.076,.182)**	.027	(-.006,.063)
		SDQ-CP (Teacher)	3561 (1157,1316)	**.140**	**(.112,.169)**	.025	(-.050,.098)	.001	(-.060,.063)
		SDQ-CP(Child)	5523 (1799,2012)	**.344**	**(.323,.364)**	**.282**	**(.223,.342)**	**.199**	**(.140,.259)**
	Five-year	SDQ-CP (Parent)	3851 (1249,1420)	**.134**	**(.109,.160)**	**.070**	**(.003,.144)**	.002	(-.047,.052)
		SDQ-CP (Child)	3807 (1241,1404)	**.174**	**(.149,.200)**	**.116**	**(.038,.189)**	.018	(-.051,.087)
Pro-social	Concurrent	SDQ-Prosocial (Parent)	5538 (1806,2013)	**-.028**	**(-.047,-.010)**	**.048**	**(.002,.095)**	.000	(-.035,.036)

© 2017 Singham T et al. *JAMA Psychiatry.*

eTable 2. Extended Findings for Phenotypic Analyses, DZ (Same-Sex), MZ Twin Differences Analyses for Peer Victimization at Concurrent, and Mental Health Outcomes at Concurrent and 16 (i.e. Concurrent and Five-Year Analyses)

		SDQ-Prosocial (Teacher)	3518 (1147,1300)	**-.075**	**(-.099,-.050)**	.016	(-.047,.077)	-.037	(-.094,.019)
		SDQ-Prosocial (Child)	5526 (1799,2013)	**-.026**	**(-.046,-.007)**	.027	(-.028,.081)	.017	(-.035,.070)
	Five-year	SDQ-Prosocial (Parent)	3848 (1248,1419)	**-.030**	**(-.052,-.008)**	.032	(-.026,.090)	.009	(-.024,.041)
		SDQ-Prosocial (Child)	3807 (1241,1404)	**-.062**	**(-.086,-.037)**	.017	(-.052,.084)	-.001	(-.069,.065)
Peer Problems	Concurrent	SDQ-Peer Problems (Parent)	5525 (1799,2010)	**.267**	**(.243,.291)**	**.257**	**(.198,.315)**	**.103**	**(.064,.145)**
		SDQ-Peer Problems (Teacher)	3555 (1155,1315)	**.175**	**(.148,.206)**	**.133**	**(.070,.201)**	**.163**	**(.101,.230)**
		SDQ-Peer Problems (Child)	5523 (1799,2012)	**.407**	**(.384,.430)**	**.408**	**(.345,.473)**	**.246**	**(.180,.315)**
	Five-year	SDQ-Peer Problems (Child)	3808 (1241,1404)	**.167**	**(.142,.193)**	**.104**	**(.037,.169)**	.064	(-.003,.130)
Psychotic-like Symptoms	Five-year	SPEQ-Paranoid Thoughts (Child)	3813 (1243,1404)	**.209**	**(.182,.235)**	**.152**	**(.086,.217)**	**.075**	**(.016,.136)**
		SPEQ-Hallucinations (Child)	3817 (1245,1408)	**.146**	**(.120,.171)**	**.080**	**(.007,.150)**	.059	(-.009,.128)
		SPEQ-Grandiosity (Child)	3813 (1242,1406)	**.044**	**(.019,.068)**	.009	(-.056,.075)	.005	(-.057,.069)
		SPEQ-Cognitive Disorganisation (Child)	3806 (1238,1405)	**.139**	**(.115,.163)**	**.124**	**(.059,.189)**	**.091**	**(.031,.150)**
		SPEQ-Anhedonia (Child)	3807 (1238,1405)	**.111**	**(.087,.134)**	.033	(-.033,.097)	.017	(-.054,.085)
		SPEQ-Negative Symptoms (Parent)	3849 (1247,1419)	**.096**	**(.071,.122)**	.002	(-.066,.073)	.023	(-.013,.058)

Note: Significant estimates are presented in **bold**. SDQ Total Difficulties includes three subscales of the SDQ: Anxiety, Hyperactivity, and Conduct Problems. A fourth scale, the Peer Problems scale, is added for the SDQ including Peer Problems.

© 2017 Singham T et al. *JAMA Psychiatry*.

eTable 3. Results From the Phenotypic Analyses, DZ (Same-Sex), and MZ Twin Differences Analyses for Victimization at Age 14 and Mental Health Outcomes at Age 16 (i.e. Two-Year Analyses)

Outcome	Variable Name	Total N (DZSS, MZ)	Phenotypic		DZ Differences		MZ Differences	
			B	95% CI	B	95% CI	B	95% CI
Total Difficulties	SDQ Total Difficulties (Child)	2353 (759,929)	**.238**	**(.205,.271)**	**.238**	**(.154,.327)**	**.106**	**(.021,.187)**
Total Difficulties (Including peer problems)	SDQ including Peer Problems (Child)	2355 (759,930)	**.282**	**(.251,.316)**	**.264**	**(.181,.350)**	**.114**	**(.032,.193)**
Anxiety & Depression	SDQ-Anxiety (Child)	2354 (759,930)	**.129**	**(.097,.161)**	**.117**	**(.034, .193)**	**.117**	**(.042,.195)**
	ARBQ-Anxiety (Parent)	2387 (767,940)	**.078**	**(.047,.112)**	**.051**	**(-.008,.112)**	**.079**	**(.015,.159)**
	CASI-Anxiety (Child)	2364 (766,930)	**.131**	**(.099,.164)**	**.132**	**(.058,.214)**	**.146**	**(.065,.220)**
	MFQ-Depression (Parent)	2385 (767,937)	**.125**	**(.092,.161)**	**.101**	**(.041,.172)**	.028	(-.060,.113)
	MFQ-Depression (Child)	2363 (764,930)	**.189**	**(.156,.223)**	**.163**	**(.094,.239)**	.069	(-.033,.161)
Hyper-activity / Inattention / Impulsiveness	SDQ-Hyperactivity (Parent)	2378 (765,937)	**.173**	**(.141,.205)**	**.112**	**(.034,.192)**	.035	(-.025,.107)
	SDQ-Hyperactivity (Child)	2354 (759,929)	**.193**	**(.161,.224)**	**.209**	**(.122,.302)**	.049	(-.040,.135)
	Conners Total (Parent)	2382 (765,938)	**.185**	**(.151,.221)**	**.097**	**(.034,.164)**	.027	(-.022,.114)
	Conners Hyp-Impul (Parent)	2381 (765,937)	**.134**	**(.100,.172)**	**.067**	**(.006,.134)**	-.002	(-.052,.072)
	Conners Inatt (Parent)	2382 (765,938)	**.185**	**(.152,.219)**	**.099**	**(.031,.169)**	.041	(-.011,.118)
Conduct Problems	SDQ-CP (Parent)	2384 (767,939)	**.155**	**(.121,.188)**	**.123**	**(.054,.198)**	.033	(-.020,.087)
	SDQ-CP (Child)	2353 (759,930)	**.222**	**(.190,.255)**	**.210**	**(.108,.314)**	.056	(-.044,.152)
Pro-social	SDQ-Prosocial (Parent)	2382 (767,938)	**-.060**	**(-.087,-.034)**	.026	(-.028,.084)	-.023	(-.067,.016)
	SDQ-Prosocial (Child)	2354 (759,930)	**-.063**	**(-.093,-.032)**	-.006	(-.078,.071)	-.058	(-.150,.036)
Peer Problems	SDQ-Peer Problems (Child)	2353 (759,929)	**.267**	**(.234,.301)**	**.196**	**(.103,.283)**	.070	(-.012,.155)
Psychotic-like Symptoms	SPEQ-Paranoid Thoughts (Child)	2362 (765,928)	**.342**	**(.308,.377)**	**.252**	**(.179,.327)**	**.241**	**(.158,.333)**
	SPEQ-Hallucinations (Child)	2363 (765,930)	**.213**	**(.179,.247)**	**.149**	**(.073,.225)**	**.119**	**(.028,.214)**
	SPEQ-Grandiosity (Child)	2360 (765,928)	**.057**	**(.026,.088)**	.075	(-.014,.159)	-.077	(-.155,.001)
	SPEQ-Cognitive Disorganisation (Child)	2360 (762,930)	**.194**	**(.165,.225)**	**.215**	**(.142,.288)**	**.146**	**(.064,.226)**
	SPEQ-Anhedonia (Child)	2359 (762,929)	**.152**	**(.121,.183)**	.069	(-.016,.151)	.047	(-.043,.135)
	SPEQ-Negative Symptoms (Parent)	2383 (767,936)	**.094**	**(.062,.127)**	-.011	(-.074,.047)	.009	(-.040,.060)

Note: Significant estimates are presented in **bold**; SDQ including Peer Problems refers to the four difficulty subscales of the SDQ: Anxiety, Hyperactivity, Conduct Problems and Peer Problems.

© 2017 Singham T et al. *JAMA Psychiatry.*

eTable 4. Consideration of Early Parenting Variables

Childhood factor	Variable Name	Total N (DZSS, MZ)	Phenotypic effects			DZ Differences			MZ Differences		
			B	95% CI		B	95% CI		B	95% CI	
Parental feelings & discipline	Smack/shout	5484 (1788, 1995)	.169	.142	.196	.148	.079	.218	.050	-.037	.135
	Explain/calm	5158 (1682, 1887)	-.023	-.049	.003	-.012	-.097	.069	.036	-.053	.129
	Parental negative feelings	5489 (1788, 1998)	.139	.115	.166	.133	.063	.201	.033	.000	.043

Note: As a sensitivity analysis, the possibility of additional non-shared environmental confounding by parenting variables was explored. Several variables that are conceptually related to other forms of early victimization were used, including harsh parenting and negative parental feelings. Parental Discipline was assessed using questions adapted from a semi-structured interview [1]. From this measure, we derived at a mean score for harsh parenting across time ("Give a smack or slap" and "Telling off or shouting", assessed at age 3, 4, 7, and 9) and a mean score for positive parenting across time ("Explain to child, or reason with child" and "Be firm and calm with child", assessed at age 3, 4 and 9). Negative parental feelings were assessed using items from the Negativity subscale of the Parental Feelings Questionnaire [2], assessed at age 3, 4, 7 and 9. Phenotypic, DZ and MZ estimates were computed following the procedure described in the manuscript for main analyses.

© 2017 Singham T et al. *JAMA Psychiatry.*

eTable 5. Descriptives for Outcome Measures for Concurrent Analyses (Victimization and Outcomes Assessed at 11 Years)

Outcome	Variable Name	Mean	SD	Skew	Kurtosis
Victimization	Peer Victimization (Child)	7.76	7.36	1.05	0.43
Total Difficulties	SDQ Total Difficulties (Parent)	5.89	4.20	1.00	1.03
	SDQ Total Difficulties (Teacher)	3.77	4.04	1.60	2.94
	SDQ Total Difficulties (Child)	7.55	4.58	0.68	0.25
Total Difficulties (Including peer problems)	SDQ including Peer Problems (Parent)	6.96	5.01	1.12	1.46
	SDQ including Peer Problems (Teacher)	4.76	4.89	1.63	3.18
	SDQ including Peer Problems (Child)	8.89	5.39	0.80	0.64
Anxiety & Depression	SDQ-Anxiety (Parent)	1.79	1.90	1.27	1.44
	SDQ-Anxiety (Teacher)	1.19	1.79	1.87	3.54
	SDQ-Anxiety (Child)	2.17	2.04	1.02	0.69
	MFQ-Depression (Parent)	1.14	2.26	3.24	13.80
	MFQ-Depression (Child)	2.28	3.29	2.14	5.30
Hyper-activity / Inattention / Impulsiveness	SDQ-Hyperactivity (Parent)	2.80	2.25	0.88	0.43
	SDQ-Hyperactivity (Teacher)	2.04	2.39	1.33	1.22
	SDQ-Hyperactivity (Child)	3.51	2.30	0.47	-0.36
	Conners Total (Parent)	9.69	8.42	1.60	3.28
	Conners Hyperactivity-Impulsivity (Parent)	4.22	4.29	1.73	3.77
	Conners Inatt (Parent)	5.48	5.05	1.38	2.08
Conduct Problems	SDQ-Conduct Problems (Parent)	1.31	1.45	1.45	2.88
	SDQ-Conduct Problems (Teacher)	0.55	1.24	3.12	11.64
	SDQ-Conduct Problems(Child)	1.88	1.65	1.12	1.40
Pro-social	SDQ-Prosocial (Parent)	8.56	1.63	-1.24	1.39
	SDQ-Prosocial (Teacher)	8.06	2.07	-0.99	0.36
	SDQ-Prosocial (Child)	7.45	1.90	-0.46	-0.43
Peer Problems	SDQ-Peer Problems (Parent)	1.07	1.48	1.87	4.13
	SDQ-Peer Problems (Teacher)	0.98	1.54	2.07	4.94
	SDQ-Peer Problems (Child)	1.33	1.56	1.66	3.39

Note: SDQ including Peer Problems refers to the four difficulty subscales of the SDQ: Anxiety, Hyperactivity, Conduct Problems and Peer Problems.

© 2017 Singham T et al. *JAMA Psychiatry*.

eTable 6. Concurrent MZ Analyses Repeated With Further Control for Preexisting Twin Differences in Mental Health Difficulties at Ages 7 and 9 Years

Outcome	Baseline 7 years	Baseline 9 years	Total N (DZSS, MZ)	MZ	95% CI	MZ baseline	95% CI
SDQ Total Difficulties (Parent)	SDQ Total Difficulties (Parent)	SDQ Total Difficulties (Parent)	5146 (1676,1892)	**.040**	**(.005,.074)**	**.032**	**(.001,.065)**
SDQ Total Difficulties (Teacher)	SDQ Total Difficulties (Teacher)	SDQ Total Difficulties (Teacher)	2834 (923,1065)	**.107**	**(.045,.171)**	**.103**	**(.039,.169)**
SDQ Total Difficulties (Child)	SDQ Total Difficulties (Parent)	SDQ Total Difficulties (Child)	5142 (1675,1890)	**.246**	**(.191,.302)**	**.240**	**(.185,.295)**
SDQ including Peer Problems (Parent)	SDQ including Peer Problems (Parent)	SDQ including Peer Problems (Parent)	5147 (1676,1892)	**.062**	**(.029,.095)**	**.052**	**(.021,.083)**
SDQ including Peer Problems (Teacher)	SDQ including Peer Problems (Teacher)	SDQ including Peer Problems (Teacher)	2853 (930,1069)	**.137**	**(.075,.199)**	**.134**	**(.072,.197)**
SDQ including Peer Problems (Child)	SDQ including Peer Problems (Parent)	SDQ including Peer Problems (Child)	5146 (1675,1893)	**.279**	**(.224,.335)**	**.272**	**(.216,.327)**
SDQ-Anxiety (Parent)	SDQ-Anxiety (Parent)	SDQ-Anxiety (Parent)	5147 (1676,1892)	.036	(-.015,.086)	.034	(-.014,.082)
SDQ-Anxiety (Teacher)	SDQ-Anxiety (Teacher)	SDQ-Anxiety (Teacher)	2837 (925,1063)	**.166**	**(.092,.242)**	**.162**	**(.087,.236)**
SDQ-Anxiety (Child)	SDQ-Anxiety (Parent)	SDQ-Anxiety (Child)	5144 (1674,1893)	**.269**	**(.209,.328)**	**.265**	**(.208,.325)**
MFQ-Depression (Parent)	SDQ-Anxiety (Parent)	SDQ-Anxiety (Parent)	5136 (1676,1891)	**.087**	**(.028,.146)**	**.087**	**(.029,.146)**
MFQ-Depression (Child)	SDQ-Anxiety (Parent)	SDQ-Anxiety (Child)	5172 (1686,1899)	**.362**	**(.300,.431)**	**.361**	**(.296,.426)**
SDQ-Hyperactivity (Parent)	SDQ-Hyperactivity (Parent)	SDQ-Hyperactivity (Parent)	5145 (1675,1892)	.025	(-.008,.061)	.020	(-.009,.050)
SDQ-Hyperactivity (Teacher)	SDQ-Hyperactivity (Teacher)	SDQ-Hyperactivity (Teacher)	2854 (932,1070)	.050	(-.013,.114)	.048	(-.014,.114)
SDQ-Hyperactivity (Child)	SDQ-Hyperactivity (Parent)	SDQ-Hyperactivity (Child)	5142 (1674,1892)	**.102**	**(.050,.157)**	**.100**	**(.046,.155)**
Conners Total (Parent)	Conners Total (Age 8)		4547 (1472,1672)	.024	(-.006,.056)	.012	(-.015,.039)
Conners Hyperactivity-Impulsivity (Parent)	Conners Hyperactivity-Impulsivity (Age 8)		4544 (1471,1672)	.005	(-.023,.034)	-.001	(-.026,.024)
Conners Inattention (Parent)	Conners Inattention (Age 8)		4544 (1471,1671)	**.035**	**(.000,.073)**	.022	(-.012,.055)
SDQ-Conduct Problems (Parent)	SDQ-Conduct Problems (Parent)	SDQ-Conduct Problems (Parent)	5149 (1678,1891)	.030	(-.004,.067)	.023	(-.012,.059)
SDQ-Conduct Problems (Teacher)	SDQ-Conduct Problems (Teacher)	SDQ-Conduct Problems (Teacher)	2854 (932,1070)	.009	(-.056,.077)	.005	(-.061,.071)
SDQ-Conduct Problems(Child)	SDQ-Conduct Problems(Parent)	SDQ-Conduct Problems(Child)	5149 (1677,1893)	**.207**	**(.146,.270)**	**.197**	**(.137,.257)**
SDQ-Prosocial (Parent)	SDQ-Prosocial (Parent)	SDQ-Prosocial (Parent)	5160 (1684,1894)	-.003	(-.039,.033)	-.011	(-.046,.022)
SDQ-Prosocial (Teacher)	SDQ-Prosocial (Teacher)	SDQ-Prosocial (Teacher)	2814 (924,1052)	-.028	(-.090,.033)	-.029	(-.091,.033)
SDQ-Prosocial (Child)	SDQ-Prosocial (Parent)	SDQ-Prosocial (Child)	5148 (1675,1893)	.011	(-.042,.065)	.011	(-.044,.064)
SDQ-Peer Problems (Parent)	SDQ-Peer Problems (Parent)	SDQ-Peer Problems (Parent)	5146 (1676,1892)	**.097**	**(.059,.139)**	**.086**	**(.048,.125)**
SDQ-Peer Problems (Teacher)	SDQ-Peer Problems (Teacher)	SDQ-Peer Problems (Teacher)	2839 (925,1067)	**.151**	**(.080,.224)**	**.153**	**(.083,.228)**
SDQ-Peer Problems (Child)	SDQ-Peer Problems (Parent)	SDQ-Peer Problems (Child)	5144 (1676,1890)	**.242**	**(.170,.314)**	**.239**	**(.164,.308)**

Note:
In MZ twin analyses, we further controlled for within-twin pairs differences in prior mental difficulties measured at age 7 and/or 9 years. Twin pairs had to have complete data on peer victimization, the respective outcome measures and prior mental health measures in order to be included in these additional analyses. In order to maximize available Ns, we averaged measures across ages 7 and 9 years. This procedure was followed for all outcome measures, except for the Conners scale, where data was collected at age 8 years instead.
Wherever possible, the corresponding baseline measures were used (e.g. SDQ parent-rated anxiety when examining the SDQ parent-rated anxiety as an outcome). When not possible, the next best option was used. Detail on measures that were used to generate this score of prior mental health difficulties aree presented in the columns labelled "Baseline 7 years" and "Baseline 9 years".
To facilitate comparison, we present MZ differences without this additional adjustment in the column “MZ”. These analyses are the same as in the manuscript but with a smaller N due to missing data on prior mental health measures.
The column "MZ baseline" contains the MZ estimates when controlling for prior mental health difficulties.
Because the "MZ" analysis and the "MZ baseline" analysis were conducted on the same sample of participants (no. of pairs can be found in the column "Total N"), any change in significance between "MZ" and "MZ baseline" is due to the baseline controls being added into the regression, and not due to a change in sample size..
All significant estimates are presented in **bold**.
SDQ including Peer Problems refers to the four difficulty subscales of the SDQ: Anxiety, Hyperactivity, Conduct Problems and Peer Problems.

© 2017 Singham T et al. *JAMA Psychiatry*.

eTable 7. Descriptives for Two-Year Analyses (Victimization Assessed at Age 14 Years and Outcomes at 16 Years)

Outcome	Variable Name	Mean	SD	Skew	Kurtosis
Victimization	Peer Victimization (Child)	7.31	7.06	1.15	0.80
Total Difficulties	SDQ Total Difficulties (Child)	7.65	4.33	0.69	0.27
Total Difficulties (Including peer problems)	SDQ including Peer Problems (Child)	9.16	5.01	0.73	0.45
Anxiety & Depression	SDQ-Anxiety (Child)	2.70	2.24	0.80	0.09
	ARBQ-Anxiety (Parent)	3.25	3.76	2.04	6.02
	CASI-Anxiety (Child)	7.84	5.74	1.21	1.78
	MFQ-Depression (Parent)	0.88	2.16	4.21	22.29
	MFQ-Depression (Child)	2.75	3.71	2.16	5.23
Hyper-activity / Inattention / Impulsiveness	SDQ-Hyperactivity (Parent)	2.05	1.83	1.19	1.54
	SDQ-Hyperactivity (Child)	3.44	2.29	0.55	-0.22
	Conners Total (Parent)	5.83	6.54	2.11	6.45
	Conners Hyperactivity-Impulsivity (Parent)	2.16	3.01	2.56	9.22
	Conners Inattention (Parent)	3.67	4.44	1.90	4.57
Conduct Problems	SDQ-Conduct Problems (Parent)	1.10	1.29	1.69	4.17
	SDQ-Conduct Problems (Child)	1.51	1.39	1.34	2.32
Pro-social	SDQ-Prosocial (Parent)	8.30	1.90	-1.12	0.78
	SDQ-Prosocial (Child)	7.27	1.90	-0.52	-0.12
Peer Problems	SDQ-Peer Problems (Child)	1.51	1.47	1.32	2.16
Psychotic-like Symptoms	SPEQ-Paranoid Thoughts (Child)	12.19	10.62	1.57	3.32
	SPEQ-Hallucinations (Child)	4.59	5.98	2.17	6.12
	SPEQ-Grandiosity (Child)	5.27	4.39	1.20	1.53
	SPEQ-Cognitive Disorganisation (Child)	3.84	2.82	0.47	-0.65
	SPEQ-Anhedonia (Child)	1.20	1.28	1.24	1.48
	SPEQ-Negative Symptoms (Parent)	2.58	3.61	2.32	7.18

Note: SDQ including Peer Problems refers to the four difficulty subscales of the SDQ: Anxiety, Hyperactivity, Conduct Problems and Peer Problems.

© 2017 Singham T et al. *JAMA Psychiatry.*

eTable 8. Two-Year MZ Analyses Repeated With Further Control for Preexisting Twin Differences in Mental Health Difficulties at Ages 7 and 9 Years

Outcome	Baseline 11 years	Total N (DZSS, MZ)	MZ	95% CI	MZ baseline	95% CI
SDQ Total Difficulties (Child)	SDQ Total Difficulties (Child)	2174 (703,853)	**.106**	**(.016,.191)**	.075	(-.013,.159)
SDQ including Peer Problems (Child)	SDQ including Peer Problems (Child)	2175 (703,854)	**.113**	**(.028,.195)**	**.083**	**(.000,.164)**
ARBQ-Anxiety (Parent)	SDQ-Anxiety (Parent)	2220 (714,868)	**.098**	**(.032,.182)**	**.079**	**(.013,.159)**
CASI-Anxiety (Child)	SDQ-Anxiety (Child)	2183 (708,854)	**.144**	**(.060,.223)**	**.131**	**(.049,.211)**
SDQ-Anxiety (Child)	SDQ-Anxiety (Child)	2175 (703,854)	**.117**	**(.034,.197)**	**.092**	**(.017,.172)**
MFQ-Depression (Parent)	SDQ-Anxiety (Parent)	2212 (714,864)	.033	(-.056,.122)	.020	(-.069,.108)
MFQ-Depression (Child)	SDQ-Anxiety (Child)	2188 (710,854)	.085	(-.020,.186)	.061	(-.041,.158)
SDQ-Hyperactivity (Parent)	SDQ-Hyperactivity (Parent)	2211 (712,865)	.040	(-.022,.114)	.034	(-.025,.101)
SDQ-Hyperactivity (Child)	SDQ-Hyperactivity (Child)	2190 (707,859)	.060	(-.029, .151)	.050	(-.042, .140)
Conners Total (Parent)	Conners Total (Parent)	2215 (711,867)	.031	(-.020,.120)	.023	(-.025,.104)
Conners Hyperactivity-Impulsivity (Parent)	Conners Hyperactivity-Impulsivity (Parent)	2213 (710,866)	.004	(-.047,.082)	.006	(-.044,.087)
Conners Inattention (Parent)	Conners Inattention (Parent)	2216 (711,867)	.043	(-.010,.127)	.027	(-.021,.087)
SDQ-Conduct Problems (Parent)	SDQ-Conduct Problems (Parent)	2216 (714,866)	.042	(-.015,.099)	.027	(-.030,.084)
SDQ-Conduct Problems (Child)	SDQ-Conduct Problems (Child)	2174 (703,854)	.044	(-.063,.141)	.017	(-.091,.116)
SDQ-Prosocial (Parent)	SDQ-Prosocial (Parent)	2215 (714,866)	-.024	(-.070,.018)	-.025	(-.072,.016)
SDQ-Prosocial (Child)	SDQ-Prosocial (Child)	2175 (703,854)	-.056	(-.150,.040)	-.048	(-.140,.046)
SDQ-Peer Problems (Child)	SDQ-Peer Problems (Child)	2174 (703,853)	.068	(-.013,.156)	.063	(-.018,.152)
SPEQ-Paranoid Thoughts (Child)	SDQ including Peer Problems (Child)	2183 (708,853)	**.225**	**(.139,.314)**	**.215**	**(.133,.309)**
SPEQ-Hallucinations (Child)	SDQ including Peer Problems (Child)	2182 (707,855)	**.104**	**(.009,.200)**	.095	(-.004,.189)
SPEQ-Grandiosity (Child)	SDQ including Peer Problems (Child)	2180 (707,852)	-.081	(-.163,.000)	-.079	(-.163,.001)
SPEQ-Cognitive Disorganisation (Child)	SDQ including Peer Problems (Child)	2178 (704,854)	**.142**	**(.056,.227)**	**.125**	**(.041,.211)**
SPEQ-Anhedonia (Child)	SDQ including Peer Problems (Child)	2177 (704,853)	.033	(-.057,.120)	.018	(-.070,.104)
SPEQ-Negative Symptoms (Parent)	SDQ including Peer Problems (Parent)	2216 (714,865)	.009	(-.042,.062)	-.003	(-.052,.050)

Note: For the two-year analyses, peer victimization was assessed at age 14 years and outcomes at Five-year years. Measures of prior mental health difficulties were therefore available at Concurrent years. Few pairs were lost when including prior mental health difficulties. See Note of eTable 6 for additional information on this set of analyses.

© 2017 Singham T et al. *JAMA Psychiatry.*

eTable 9. Descriptives for Five-Year Analyses (Victimization Assessed at Age 11 Years and Outcomes at 16 Years)

Outcome	Variable Name	Mean	SD	Skew	Kurtosis
Victimization	Peer Victimization (Child)	7.47	7.21	1.11	0.62
Total Difficulties	SDQ Total Difficulties (Child)	7.85	4.41	0.64	0.17
Total Difficulties (Including peer problems)	SDQ including Peer Problems (Child)	9.37	5.06	0.67	0.32
Anxiety & Depression	SDQ-Anxiety (Child)	2.73	2.23	0.75	0.00
	ARBQ-Anxiety (Parent)	3.39	3.97	2.07	6.00
	CASI-Anxiety (Child)	7.92	5.81	1.15	1.46
	MFQ-Depression (Parent)	0.93	2.18	3.99	20.22
	MFQ-Depression (Child)	2.74	3.68	2.20	5.57
Hyper-activity / Inattention / Impulsiveness	SDQ-Hyperactivity (Parent)	2.17	1.89	1.16	1.52
	SDQ-Hyperactivity (Child)	3.52	2.30	0.50	-0.31
	Conners Total (Parent)	6.33	6.88	2.02	5.68
	Conners Hyperactivity-Impulsivity (Parent)	2.38	3.22	2.49	8.51
	Conners Inattention (Parent)	3.95	4.60	1.78	3.87
Conduct Problems	SDQ-Conduct Problems (Parent)	1.18	1.35	1.64	3.91
	SDQ-Conduct Problems (Child)	1.61	1.45	1.31	2.14
Pro-social	SDQ-Prosocial (Parent)	8.26	1.92	-1.13	0.81
	SDQ-Prosocial (Child)	7.17	1.94	-0.48	-0.19
Peer Problems	SDQ-Peer Problems (Child)	1.51	1.48	1.35	2.38
Psychotic-like Symptoms	SPEQ-Paranoid Thoughts (Child)	12.15	10.56	1.57	3.36
	SPEQ-Hallucinations (Child)	4.65	6.02	2.12	5.67
	SPEQ-Grandiosity (Child)	5.32	4.44	1.18	1.40
	SPEQ-Cognitive Disorganisation (Child)	3.92	2.84	0.47	-0.67
	SPEQ-Anhedonia (Child)	1.26	1.30	1.16	1.30
	SPEQ-Negative Symptoms (Parent)	2.71	3.65	2.24	6.59

Note: SDQ including Peer Problems refers to the four difficulty subscales of the SDQ: Anxiety, Hyperactivity, Conduct Problems and Peer Problems.

© 2017 Singham T et al. *JAMA Psychiatry.*

eTable 10. Five-Year MZ Analyses Repeated With Further Control for Preexisting Twin Differences in Mental Health Difficulties at Ages 7 and 9 Years

Outcome	Baseline 7 years	Baseline 9 years	Total N (DZSS, MZ)	MZ	95% CI	MZ baseline	95% CI
SDQ Total Difficulties (Child)	SDQ Total Difficulties (Parent)	SDQ Total Difficulties (Child)	3598 (1170,1332)	**.062**	**(.002,.123)**	**.059**	**(-.001,.121)**
SDQ including Peer Problems (Child)	SDQ including Peer Problems (Parent)	SDQ including Peer Problems (Child)	3599 (1170,1333)	**.069**	**(.008,.129)**	**.065**	**(.006,.125)**
ARBQ-Anxiety (Parent)	SDQ-Anxiety (Parent)	SDQ-Anxiety (Parent)	3640 (1177,1350)	.031	(-.025,.084)	.029	(-.025,.081)
CASI-Anxiety (Child)	SDQ-Anxiety (Parent)	SDQ-Anxiety (Child)	3609 (1175,1336)	.013	(-.048,.071)	.013	(-.047,.072)
SDQ-Anxiety (Child)	SDQ-Anxiety (Parent)	SDQ-Anxiety (Child)	3600 (1170,1334)	.041	(-.020,.100)	.037	(-.024,.095)
MFQ-Depression (Parent)	SDQ-Anxiety (Parent)	SDQ-Anxiety (Parent)	3637 (1177,1347)	-.029	(-.104,.028)	-.030	(-.104,.026)
MFQ-Depression (Child)	SDQ-Anxiety (Parent)	SDQ-Anxiety (Child)	3608 (1173,1338)	.031	(-.040,.103)	.031	(-.040,.101)
SDQ-Hyperactivity (Parent)	SDQ-Hyperactivity (Parent)	SDQ-Hyperactivity (Parent)	3629 (1174,1347)	.032	(-.013,.082)	.032	(-.011,.076)
SDQ-Hyperactivity (Child)	SDQ-Hyperactivity (Parent)	SDQ-Hyperactivity (Child)	3595 (1169,1331)	.062	(-.005, .133)	.065	(.000, .134)
Conners Total (Parent)	Conners Total (Age 8)		3311 (1026,1230)	.027	(-.011,.075)	.022	(-.015,.067)
Conners Hyperactivity-Impulsivity (Parent)	Conners Hyperactivity-Impulsivity (Age 8)		3307 (1062,1229)	-.003	(-.040,.036)	-.004	(-.040,.033)
Conners Inatt (Parent)	Conners Inattention (Age 8)		3309 (1061,1230)	.042	(-.003,.098)	.034	(-.008,.084)
SDQ-Conduct Problems (Parent)	SDQ-Conduct Problems (Parent)	SDQ-Conduct Problems (Parent)	3639 (1179,1349)	.000	(-.051,.051)	-.002	(-.053,.048)
SDQ-Conduct Problems (Child)	SDQ-Conduct Problems(Parent)	SDQ-Conduct Problems(Child)	3600 (1172,1333)	.024	(-.045,.095)	.021	(-.048,.092)
SDQ-Prosocial (Parent)	SDQ-Prosocial (Parent)	SDQ-Prosocial (Parent)	3635 (1177,1348)	.009	(-.025,.044)	.002	(-.031,.037)
SDQ-Prosocial (Child)	SDQ-Prosocial (Parent)	SDQ-Prosocial (Child)	3598 (1170,1332)	-.015	(-.086,.052)	-.017	(-.088,.052)
SDQ-Peer Problems (Child)	SDQ-Peer Problems (Parent)	SDQ-Peer Problems (Child)	3599 (1170,1333)	.059	(-.008,.124)	.059	(-.006,.127)
SPEQ-Paranoid Thoughts (Child)	SDQ including Peer Problems (Parent)	SDQ including Peer Problems (Child)	3604 (1172,1334)	**.082**	**(.022,.144)**	**.085**	**(.023,.147)**
SPEQ-Hallucinations (Child)	SDQ including Peer Problems (Parent)	SDQ including Peer Problems (Child)	3607 (1174,1337)	.061	(-.005,.135)	.061	(-.006,.136)
SPEQ-Grandiosity (Child)	SDQ including Peer Problems (Parent)	SDQ including Peer Problems (Child)	3604 (1171,1336)	.011	(-.050,.078)	.012	(-.051,.076)
SPEQ-Cognitive Disorganisation (Child)	SDQ including Peer Problems (Parent)	SDQ including Peer Problems (Child)	3598 (1168,1334)	**.087**	**(.026,.147)**	**.084**	**(.023,.144)**
SPEQ-Anhedonia (Child)	SDQ including Peer Problems (Parent)	SDQ including Peer Problems (Child)	3598 (1167,1334)	.008	(-.065,.078)	.006	(-.064,.077)
SPEQ-Negative Symptoms (Parent)	SDQ including Peer Problems (Parent)	SDQ including Peer Problems (Parent)	3636 (1175,1349)	.014	(-.023,.048)	.012	(-.025,.047)

Note: See Note for eTable 6.

© 2017 Singham T et al. *JAMA Psychiatry.*

eTable 11. Concurrent Analyses With Physical Victimization

Variable	Phenotypic		DZ Differences		MZ Differences	
	B	95% CI	B	95% CI	B	95% CI
SDQ Total Difficulties (Parent)	**.197**	**(.177,.217)**	**.149**	**(.096,.201)**	**.032**	**(.002,.063)**
SDQ Total Difficulties (Teacher)	**.199**	**(.171,.228)**	**.034**	**(-.038,.110)**	**.089**	**(.032,.146)**
SDQ Total Difficulties (Child)	**.306**	**(.285,.326)**	**.252**	**(.194,.308)**	**.116**	**(.067,.167)**
SDQ including Peer Problems (Parent)	**.227**	**(.207,.248)**	**.186**	**(.134,.236)**	**.053**	**(.021,.083)**
SDQ including Peer Problems (Teacher)	**.214**	**(.184,.244)**	.059	(-.015,.134)	**.120**	**(.063,.177)**
SDQ including Peer Problems (Child)	**.351**	**(.331,.371)**	**.299**	**(.242,.355)**	**.143**	**(.094,.198)**
SDQ-Anxiety (Parent)	**.080**	**(.060,.101)**	**.068**	**(.015,.120)**	.040	(-.004,.083)
SDQ-Anxiety (Teacher)	**.110**	**(.082,.139)**	**.035**	**(-.041,.112)**	**.133**	**(.065,.199)**
SDQ-Anxiety (Child)	**.207**	**(.187,.229)**	**.215**	**(.159,.270)**	**.140**	**(.087,.191)**
MFQ-Depression (Parent)	**.149**	**(.126,.171)**	**.163**	**(.106,.223)**	**.072**	**(.021,.123)**
MFQ-Depression (Child)	**.311**	**(.288,.334)**	**.302**	**(.238,.364)**	**.203**	**(.150,.260)**
SDQ-Hyperactivity (Parent)	**.218**	**(.198,.239)**	**.157**	**(.098,.218)**	.012	(-.017,.042)
SDQ-Hyperactivity (Teacher)	**.182**	**(.155,.208)**	.042	(-.028,.115)	.034	(-.020,.089)
SDQ-Hyperactivity (Child)	**.235**	**(.214,.254)**	**.175**	**(.115,.233)**	.025	(-.024,.074)
Conners Total (Parent)	**.218**	**(.197,.240)**	**.153**	**(.099,.212)**	.008	(-.018,.036)
Conners Hyperactivity-Impusivity (Parent)	**.175**	**(.153,.197)**	**.117**	**(.062,.174)**	-.008	(-.032,.017)
Conners Inattention (Parent)	**.219**	**(.198,.240)**	**.156**	**(.100,.216)**	.021	(-.010,.054)
SDQ-Conduct Problems (Parent)	**.151**	**(.131,.173)**	**.097**	**(.044,.155)**	.024	(-.009,.058)
SDQ-Conduct Problems (Teacher)	**.147**	**(.119,.178)**	-.001	(-.079,.076)	.023	(-.031,.077)
SDQ-Conduct Problems(Child)	**.282**	**(.261,.304)**	**.190**	**(.126,.253)**	**.115**	**(.061,.169)**
SDQ-Prosocial (Parent)	**-.050**	**(-.069,-.032)**	.039	(-.008,.086)	-.002	(-.036,.032)
SDQ-Prosocial (Teacher)	**-.110**	**(-.135,-.085)**	.013	(-.049,.080)	-.053	(-.104,-.002)
SDQ-Prosocial (Child)	**-.071**	**(-.090,-.051)**	.009	(-.046,.062)	.034	(-.014,.083)
SDQ-Peer Problems (Parent)	**.224**	**(.199,.248)**	**.205**	**(.140,.267)**	**.087**	**(.050,.126)**
SDQ-Peer Problems (Teacher)	**.162**	**(.133,.194)**	**.095**	**(.022,.172)**	**.151**	**(.093,.213)**
SDQ-Peer Problems (Child)	**.324**	**(.300,.348)**	**.290**	**(.227,.356)**	**.154**	**(.090,.221)**

Note: Significant estimates are presented in **bold**. SDQ including Peer Problems refers to the four difficulty subscales of the SDQ: Anxiety, Hyperactivity, Conduct Problems and Peer Problems.

© 2017 Singham T et al. *JAMA Psychiatry.*

eTable 12. Five-Year Analyses With Physical Victimization

Variable	Phenotypic		DZ Differences		MZ Differences	
	B	95% CI	B	95% CI	B	95% CI
SDQ Total Difficulties (Child)	**.097**	**(.073,.121)**	**.061**	**(-.003,.131)**	**.056**	**(.002,.113)**
SDQ including Peer Problems (Child)	**.119**	**(.096,.144)**	**.072**	**(.006,.134)**	**.067**	**(.014,.124)**
ARBQ-Anxiety (Parent)	.002	(-.022,.025)	.027	(-.029,.087)	.001	(-.047,.044)
CASI-Anxiety (Child)	.005	(-.018,.030)	**.075**	**(.016,.137)**	.019	(-.040,.077)
SDQ-Anxiety (Child)	**-.028**	**(-.052,-.004)**	.031	(-.029,.091)	.046	(-.008,.100)
MFQ-Depression (Parent)	**.042**	**(.018,.069)**	.034	(-.029,.113)	-.043	(-.100,.003)
MFQ-Depression (Child)	**.043**	**(.019,.067)**	**.076**	**(.013,.134)**	.030	(-.030,.088)
SDQ-Hyperactivity (Parent)	**.172**	**(.146,.198)**	**.082**	**(.005,.162)**	.013	(-.026,.052)
SDQ-Hyperactivity (Child)	**.135**	**(.110, .158)**	.049	(-.021, .122)	.049	(-.007, .106)
Conners Total (Parent)	**.171**	**(.145,.198)**	**.080**	**(.015,.151)**	.018	(-.014,.051)
Conners Hyperactivity-Impulsivity (Parent)	**.119**	**(.092,.149)**	**.105**	**(.037,.185)**	-.002	(-.035,.031)
Conners Inattention (Parent)	**.179**	**(.153,.206)**	.046	(-.029,.119)	.028	(-.006,.067)
SDQ-Conduct Problems (Parent)	**.104**	**(.079,.128)**	.048	(-.018,.119)	-.003	(-.046,.038)
SDQ-Conduct Problems (Child)	**.142**	**(.117,.167)**	.058	(-.026,.141)	.022	(-.040,.084)
SDQ-Prosocial (Parent)	**-.056**	**(-.079,-.035)**	.016	(-.043,.075)	-.010	(-.041,.020)
SDQ-Prosocial (Child)	**-.100**	**(-.125,-.076)**	-.016	(-.083,.052)	.017	(-.049,.079)
SDQ-Peer Problems (Child)	**.123**	**(.098,.149)**	**.062**	**(-.007,.131)**	**.063**	**(.002,.124)**
SPEQ-Paranoid Thoughts (Child)	**.128**	**(.102,.154)**	**.085**	**(.020,.149)**	**.062**	**(.003,.122)**
SPEQ-Hallucinations (Child)	**.085**	**(.061,.111)**	.029	(-.041,.095)	.054	(-.007,.121)
SPEQ-Grandiosity (Child)	**.065**	**(.042,.089)**	.054	(-.010,.117)	**.059**	**(.004,.118)**
SPEQ-Cognitive Disorganisation (Child)	**.060**	**(.037,.085)**	.057	(-.005,.123)	**.097**	**(.045,.152)**
SPEQ-Anhedonia (Child)	**.109**	**(.086,.133)**	.060	(-.005,.121)	.021	(-.035,.080)
SPEQ-Negative Symptoms (Parent)	**.082**	**(.058,.108)**	-.005	(-.074,.062)	.009	(-.025,.038)

Note: Significant estimates are presented in **bold**. SDQ including Peer Problems refers to the four difficulty subscales of the SDQ: Anxiety, Hyperactivity, Conduct Problems and Peer Problems.

© 2017 Singham T et al. *JAMA Psychiatry.*

eTable 13. Two-Year Analyses With Physical Victimization

Variable	Phenotypic		DZ Differences		MZ Differences	
	B	95% CI	B	95% CI	B	95% CI
SDQ Total Difficulties (Child)	**.137**	**(.105,.170)**	**.194**	**(.106,.287)**	.046	(-.047,.138)
SDQ including Peer Problems (Child)	**.176**	**(.143,.209)**	**.205**	**(.121,.297)**	.057	(-.028,.153)
ARBQ-Anxiety (Parent)	**.015**	**(-.016,.046)**	.018	(-.062,.087)	**.061**	**(.004,.131)**
CASI-Anxiety (Child)	-.005	(-.035,.030)	**.080**	**(.002,.160)**	.049	(-.033,.136)
SDQ-Anxiety (Child)	-.006	(-.036,.026)	.062	(-.020,.137)	.008	(-.075,.086)
MFQ-Depression (Parent)	**.073**	**(.039,.110)**	**.078**	**(.010,.183)**	.037	(-.035,.114)
MFQ-Depression (Child)	**.070**	**(.038,.105)**	**.092**	**(.022,.163)**	-.001	(-.098,.101)
SDQ-Hyperactivity (Parent)	**.182**	**(.149,.216)**	**.115**	**(.033,.209)**	.042	(-.022,.113)
SDQ-Hyperactivity (Child)	**.159**	**(.129, .191)**	**.206**	**(.117, .304)**	.069	(-.021, .157)
Conners Total (Parent)	**.193**	**(.158,.232)**	**.097**	**(.031,.189)**	.042	(-.007,.120)
Conners Hyperactivity-Impulsivity (Parent)	**.134**	**(.097,.179)**	**.091**	**(.027,.191)**	.040	(-.010,.103)
Conners Inattention (Parent)	**.196**	**(.161,.233)**	**.081**	**(.010,.159)**	.035	(-.021,.110)
SDQ-Conduct Problems (Parent)	**.134**	**(.100,.170)**	**.127**	**(.047,.215)**	.042	(-.012,.101)
SDQ-Conduct Problems (Child)	**.182**	**(.148,.217)**	**.166**	**(.063,.268)**	.013	(-.093,.119)
SDQ-Prosocial (Parent)	**-.078**	**(-.108,-.051)**	.040	(-.019,.103)	-.030	(-.083,.013)
SDQ-Prosocial (Child)	**-.122**	**(-.153,-.091)**	-.029	(-.108,.046)	-.036	(-.134,.065)
SDQ-Peer Problems (Child)	**.198**	**(.164,.234)**	**.127**	**(.038,.211)**	.052	(-.043,.147)
SPEQ-Paranoid Thoughts (Child)	**.210**	**(.175,.247)**	**.140**	**(.065,.219)**	**.163**	**(.072,.263)**
SPEQ-Hallucinations (Child)	**.115**	**(.081,.151)**	**.098**	**(.012,.180)**	.027	(-.089,.122)
SPEQ-Grandiosity (Child)	**.071**	**(.040,.104)**	.076	(-.010,.165)	-.061	(-.149,.020)
SPEQ-Cognitive Disorganisation (Child)	**.076**	**(.047,.105)**	**.158**	**(.085,.234)**	.052	(-.029,.132)
SPEQ-Anhedonia (Child)	**.138**	**(.105,.169)**	.010	(-.076,.098)	.038	(-.063,.132)
SPEQ-Negative Symptoms (Parent)	**.100**	**(.067,.135)**	-.042	(-.127,.030)	-.007	(-.053,.043)

Note: Significant estimates are presented in **bold**. SDQ including Peer Problems refers to the four difficulty subscales of the SDQ: Anxiety, Hyperactivity, Conduct Problems and Peer Problems.

© 2017 Singham T et al. *JAMA Psychiatry*.

eTable 14. Concurrent Analyses With Verbal Victimization

Variable	Phenotypic		DZ Differences		MZ Differences	
	B	95% CI	B	95% CI	B	95% CI
SDQ Total Difficulties (Parent)	**.202**	**(.183,.221)**	**.146**	**(.101,.193)**	.028	(-.004,.058)
SDQ Total Difficulties (Teacher)	**.177**	**(.151,.203)**	**.098**	**(.037,.163)**	.042	(-.010,.094)
SDQ Total Difficulties (Child)	**.352**	**(.332,.371)**	**.300**	**(.249,.352)**	**.177**	**(.127,.226)**
SDQ including Peer Problems (Parent)	**.239**	**(.220,.259)**	**.196**	**(.152,.240)**	**.047**	**(.017,.077)**
SDQ including Peer Problems (Teacher)	**.197**	**(.171,.223)**	**.114**	**(.053,.177)**	**.077**	**(.027,.129)**
SDQ including Peer Problems (Child)	**.405**	**(.386,.424)**	**.364**	**(.314,.412)**	**.212**	**(.164,.260)**
SDQ-Anxiety (Parent)	**.134**	**(.114,.154)**	**.136**	**(.083,.189)**	**.064**	**(.018,.111)**
SDQ-Anxiety (Teacher)	**.141**	**(.115,.167)**	**.114**	**(.041,.188)**	**.108**	**(.045,.169)**
SDQ-Anxiety (Child)	**.296**	**(.277,.317)**	**.279**	**(.221,.335)**	**.214**	**(.163,.267)**
MFQ-Depression (Parent)	**.172**	**(.151,.193)**	**.168**	**(.119,.224)**	**.075**	**(.027,.128)**
MFQ-Depression (Child)	**.373**	**(.351,.395)**	**.382**	**(.327,.443)**	**.280**	**(.222,.337)**
SDQ-Hyperactivity (Parent)	**.184**	**(.164,.205)**	**.101**	**(.046,.157)**	-.006	(-.038,.026)
SDQ-Hyperactivity (Teacher)	**.138**	**(.113,.162)**	.060	(-.002,.124)	-.008	(-.059,.046)
SDQ-Hyperactivity (Child)	**.235**	**(.215,.254)**	**.180**	**(.128,.232)**	**.063**	**(.016,.112)**
Conners Total (Parent)	**.190**	**(.171,.210)**	**.134**	**(.091,.179)**	.016	(-.011,.043)
Conners Hyperactivity-Impusivity (Parent)	**.156**	**(.137,.176)**	**.127**	**(.083,.171)**	.005	(-.022,.030)
Conners Inattention (Parent)	**.190**	**(.169,.210)**	**.115**	**(.066,.167)**	.022	(-.009,.055)
SDQ-Conduct Problems (Parent)	**.152**	**(.133,.172)**	**.084**	**(.040,.130)**	.006	(-.026,.037)
SDQ-Conduct Problems (Teacher)	**.118**	**(.092,.144)**	.036	(-.027,.101)	-.002	(-.055,.053)
SDQ-Conduct Problems(Child)	**.296**	**(.276,.315)**	**.238**	**(.184,.293)**	**.141**	**(.088,.195)**
SDQ-Prosocial (Parent)	**-.028**	**(-.047,-.010)**	**.052**	**(.006,.098)**	.001	(-.035,.034)
SDQ-Prosocial (Teacher)	**-.070**	**(-.094,-.046)**	.007	(-.050,.063)	-.008	(-.060,.043)
SDQ-Prosocial (Child)	**-.038**	**(-.058,-.019)**	.020	(-.032,.071)	-.007	(-.057,.042)
SDQ-Peer Problems (Parent)	**.250**	**(.229,.272)**	**.250**	**(.197,.304)**	**.082**	**(.049,.115)**
SDQ-Peer Problems (Teacher)	**.170**	**(.144,.197)**	**.106**	**(.044,.170)**	**.137**	**(.087,.192)**
SDQ-Peer Problems (Child)	**.375**	**(.354,.398)**	**.376**	**(.318,.435)**	**.211**	**(.152,.273)**

Note: Significant estimates are presented in **bold**. SDQ including Peer Problems refers to the four difficulty subscales of the SDQ: Anxiety, Hyperactivity, Conduct Problems and Peer Problems.

© 2017 Singham T et al. *JAMA Psychiatry*.

eTable 15. Five-Year Analyses With Verbal Victimization

Variable	Phenotypic		DZ Differences		MZ Differences	
	B	95% CI	B	95% CI	B	95% CI
SDQ Total Difficulties (Child)	**.161**	**(.137,.184)**	**.132**	**(.074,.192)**	.031	(-.023,.088)
SDQ including Peer Problems (Child)	**.187**	**(.163,.210)**	**.149**	**(.090,.208)**	.043	(-.014,.100)
ARBQ-Anxiety (Parent)	**.065**	**(.042,.089)**	**.052**	**(.000,.109)**	**.084**	**(.034,.142)**
CASI-Anxiety (Child)	**.091**	**(.067,.117)**	**.108**	**(.048,.170)**	.010	(-.050,.071)
SDQ-Anxiety (Child)	**.084**	**(.060,.108)**	**.083**	**(.022,.145)**	.028	(-.032,.089)
MFQ-Depression (Parent)	**.094**	**(.067,.120)**	**.082**	**(.021,.152)**	.050	(-.007,.120)
MFQ-Depression (Child)	**.121**	**(.100,.147)**	**.107**	**(.041,.173)**	.037	(-.036,.104)
SDQ-Hyperactivity (Parent)	**.152**	**(.127,.176)**	**.117**	**(.051,.182)**	.036	(-.005,.076)
SDQ-Hyperactivity (Child)	**.142**	**(.120, .166)**	**.101**	**(.036, .167)**	.033	(-.025.092)
Conners Total (Parent)	**.163**	**(.140,.188)**	**.111**	**(.058,.168)**	.019	(-.015,.056)
Conners Hyperactivity-Impulsivity (Parent)	**.122**	**(.098,.147)**	**.124**	**(.068,.185)**	.003	(-.034,.038)
Conners Inattention (Parent)	**.164**	**(.141,.188)**	**.079**	**(.015,.139)**	.025	(-.012,.065)
SDQ-Conduct Problems (Parent)	**.125**	**(.102,.149)**	**.071**	**(.013,.132)**	.007	(-.036,.050)
SDQ-Conduct Problems (Child)	**.152**	**(.127,.176)**	**.115**	**(.046,.184)**	-.004	(-.067,.060)
SDQ-Prosocial (Parent)	**-.023**	**(-.045,-.002)**	**.057**	**(.009,.108)**	.007	(-.025,.038)
SDQ-Prosocial (Child)	**-.058**	**(-.082,-.034)**	.035	(-.027,.096)	-.010	(-.072,.051)
SDQ-Peer Problems (Child)	**.168**	**(.143,.192)**	**.117**	**(.053,.181)**	.060	(-.002,.121)
SPEQ-Paranoid Thoughts (Child)	**.202**	**(.178,.228)**	**.154**	**(.094,.214)**	.039	(-.014,.096)
SPEQ-Hallucinations (Child)	**.124**	**(.099,.148)**	**.069**	**(.005,.135)**	-.002	(-.068,.061)
SPEQ-Grandiosity (Child)	**.028**	**(.004,.052)**	-.003	(-.065,.062)	-.019	(-.074,.035)
SPEQ-Cognitive Disorganisation (Child)	**.130**	**(.105,.154)**	**.114**	**(.048,.180)**	.046	(-.010,.102)
SPEQ-Anhedonia (Child)	**.097**	**(.073,.121)**	.003	(-.059,.066)	.007	(-.061,.072)
SPEQ-Negative Symptoms (Parent)	**.086**	**(.062,.110)**	-.014	(-.069,.045)	**.041**	**(.007,.080)**

Note: Significant estimates are presented in **bold**. SDQ including Peer Problems refers to the four difficulty subscales of the SDQ: Anxiety, Hyperactivity, Conduct Problems and Peer Problems.

© 2017 Singham T et al. *JAMA Psychiatry.*

eTable 16. Two-Year Analyses With Verbal Victimization

Variable	Phenotypic		DZ Differences		MZ Differences	
	B	95% CI	B	95% CI	B	95% CI
SDQ Total Difficulties (Child)	**.206**	**(.176,.237)**	**.217**	**(.133,.301)**	**.073**	**(.002,.144)**
SDQ including Peer Problems (Child)	**.243**	**(.212,.273)**	**.240**	**(.160,.323)**	**.079**	**(.008,.148)**
ARBQ-Anxiety (Parent)	**.068**	**(.040,.097)**	.051	(-.006,.108)	.041	(-.016,.097)
CASI-Anxiety (Child)	**.123**	**(.093,.154)**	**.097**	**(.022,.173)**	**.145**	**(.074,.213)**
SDQ-Anxiety (Child)	**.126**	**(.095,.158)**	**.137**	**(.049,.221)**	**.091**	**(.018,.166)**
MFQ-Depression (Parent)	**.101**	**(.070,.132)**	**.108**	**(.043,.185)**	.008	(-.079,.080)
MFQ-Depression (Child)	**.176**	**(.145,.207)**	**.162**	**(.092,.234)**	.054	(-.038,.139)
SDQ-Hyperactivity (Parent)	**.133**	**(.103,.163)**	.069	(-.006,.147)	-.011	(-.058,.039)
SDQ-Hyperactivity (Child)	**.154**	**(.124, .184)**	**.166**	**(.080, .254)**	-.003	(-.075, .070)
Conners Total (Parent)	**.141**	**(.114,.170)**	**.078**	**(.019,.141)**	-.013	(-.051,.027)
Conners Hyperactivity-Impulsivity (Parent)	**.098**	**(.070,.127)**	**.066**	**(.008,.129)**	**-.039**	**(-.081,-.001)**
Conners Inattention (Parent)	**.146**	**(.117,.177)**	**.071**	**(.006,.139)**	.007	(-.034,.049)
SDQ-Conduct Problems (Parent)	**.133**	**(.103,.163)**	**.096**	**(.028,.170)**	.040	(-.011,.095)
SDQ-Conduct Problems (Child)	**.191**	**(.160,.223)**	**.180**	**(.093,.266)**	.081	(-.003,.166)
SDQ-Prosocial (Parent)	**-.053**	**(-.078,-.029)**	.012	(-.039,.063)	-.012	(-.050,.022)
SDQ-Prosocial (Child)	**-.061**	**(-.092,-.031)**	-.010	(-.079,.060)	-.035	(-.119,.048)
SDQ-Peer Problems (Child)	**.225**	**(.194,.256)**	**.179**	**(.096,.258)**	.052	(-.020,.126)
SPEQ-Paranoid Thoughts (Child)	**.313**	**(.282,.344)**	**.229**	**(.156,.303)**	**.176**	**(.101,.257)**
SPEQ-Hallucinations (Child)	**.186**	**(.156,.217)**	**.127**	**(.053,.201)**	.069	(-.013,.152)
SPEQ-Grandiosity (Child)	**.033**	**(.003,.063)**	.064	(-.014,.140)	**-.084**	**(-.164,-.005)**
SPEQ-Cognitive Disorganisation (Child)	**.163**	**(.133,.193)**	**.170**	**(.092,.250)**	**.108**	**(.035,.180)**
SPEQ-Anhedonia (Child)	**.119**	**(.089,.149)**	.079	(-.005,.161)	.041	(-.036,.118)
SPEQ-Negative Symptoms (Parent)	**.077**	**(.048,.105)**	-.005	(-.062,.054)	.016	(-.028,.056)

Note: Significant estimates are presented in **bold**. SDQ including Peer Problems refers to the four difficulty subscales of the SDQ: Anxiety, Hyperactivity, Conduct Problems and Peer Problems.

© 2017 Singham T et al. *JAMA Psychiatry*.

eTable 17. Concurrent Analyses With Social Victimization

Variable	Phenotypic		DZ Differences		MZ Differences	
	B	95% CI	B	95% CI	B	95% CI
SDQ Total Difficulties (Parent)	**.168**	**(.148,.187)**	**.150**	**(.105,.194)**	.023	(-.006,.053)
SDQ Total Difficulties (Teacher)	**.143**	**(.117,.169)**	**.105**	**(.042,.175)**	**.055**	**(.004,.103)**
SDQ Total Difficulties (Child)	**.327**	**(.307,.346)**	**.278**	**(.227,.330)**	**.214**	**(.167,.260)**
SDQ including Peer Problems (Parent)	**.189**	**(.170,.209)**	**.175**	**(.132,.218)**	**.033**	**(.004,.061)**
SDQ including Peer Problems (Teacher)	**.148**	**(.123,.175)**	**.123**	**(.063,.185)**	**.071**	**(.022,.120)**
SDQ including Peer Problems (Child)	**.364**	**(.344,.384)**	**.322**	**(.270,.375)**	**.231**	**(.186,.280)**
SDQ-Anxiety (Parent)	**.123**	**(.104,.144)**	**.084**	**(.032,.135)**	.008	(-.033,.049)
SDQ-Anxiety (Teacher)	**.122**	**(.096,.148)**	**.108**	**(.040,.176)**	**.113**	**(.052,.172)**
SDQ-Anxiety (Child)	**.301**	**(.280,.322)**	**.231**	**(.174,.288)**	**.226**	**(.171,.279)**
MFQ-Depression (Parent)	**.151**	**(.129,.173)**	**.130**	**(.080,.186)**	**.050**	**(.008,.090)**
MFQ-Depression (Child)	**.359**	**(.336,.383)**	**.336**	**(.277,.395)**	**.299**	**(.242,.356)**
SDQ-Hyperactivity (Parent)	**.137**	**(.117,.158)**	**.127**	**(.074,.178)**	.021	(-.010,.053)
SDQ-Hyperactivity (Teacher)	**.106**	**(.081,.131)**	**.082**	**(.021,.146)**	.019	(-.030,.066)
SDQ-Hyperactivity (Child)	**.202**	**(.182,.222)**	**.179**	**(.127,.231)**	**.102**	**(.056,.149)**
Conners Total (Parent)	**.148**	**(.128,.169)**	**.137**	**(.096,.181)**	.022	(-.002,.049)
Conners Hyperactivity-Impusivity (Parent)	**.136**	**(.116,.156)**	**.141**	**(.098,.185)**	.004	(-.017,.028)
Conners Inattention (Parent)	**.139**	**(.118,.159)**	**.109**	**(.062,.158)**	**.033**	**(.003,.065)**
SDQ-Conduct Problems (Parent)	**.145**	**(.124,.165)**	**.126**	**(.082,.174)**	.026	(-.005,.057)
SDQ-Conduct Problems (Teacher)	**.093**	**(.067,.120)**	.027	(-.040,.097)	-.021	(-.071,.030)
SDQ-Conduct Problems(Child)	**.268**	**(.248,.288)**	**.238**	**(.185,.292)**	**.170**	**(.118,.222)**
SDQ-Prosocial (Parent)	**.021**	**(.003,.038)**	.037	(-.002,.078)	.001	(-.028,.032)
SDQ-Prosocial (Teacher)	-.001	(-.025,.023)	.029	(-.026,.087)	-.020	(-.072,.032)
SDQ-Prosocial (Child)	**.045**	**(.025,.063)**	.032	(-.018,.082)	.002	(-.042,.047)
SDQ-Peer Problems (Parent)	**.181**	**(.158,.203)**	**.167**	**(.116,.218)**	**.045**	**(.012,.081)**
SDQ-Peer Problems (Teacher)	**.103**	**(.077,.130)**	**.120**	**(.065,.178)**	**.080**	**(.027,.136)**
SDQ-Peer Problems (Child)	**.307**	**(.284,.330)**	**.295**	**(.235,.357)**	**.172**	**(.112,.231)**

Note: Significant estimates are presented in **bold**. SDQ including Peer Problems refers to the four difficulty subscales of the SDQ: Anxiety, Hyperactivity, Conduct Problems and Peer Problems.

© 2017 Singham T et al. *JAMA Psychiatry*.

eTable 18. Five-Year Analyses With Social Victimization

Variable	Phenotypic		DZ Differences		MZ Differences	
	B	95% CI	B	95% CI	B	95% CI
SDQ Total Difficulties (Child)	**.179**	**(.155,.204)**	**.152**	**(.092,.214)**	**.062**	**(.007,.117)**
SDQ including Peer Problems (Child)	**.188**	**(.164,.213)**	**.153**	**(.095,.211)**	**.061**	**(.007,.116)**
ARBQ-Anxiety (Parent)	**.074**	**(.050,.099)**	.036	(-.017,.084)	.001	(-.044,.044)
CASI-Anxiety (Child)	**.139**	**(.114,.164)**	**.121**	**(.057,.185)**	.020	(-.030,.070)
SDQ-Anxiety (Child)	**.117**	**(.093,.142)**	**.077**	**(.020,.135)**	.026	(-.032,.084)
MFQ-Depression (Parent)	**.102**	**(.075,.128)**	.028	(-.026,.080)	-.026	(-.085,.027)
MFQ-Depression (Child)	**.132**	**(.110,.157)**	**.070**	**(.006,.130)**	.043	(-.021,.110)
SDQ-Hyperactivity (Parent)	**.119**	**(.094,.144)**	**.095**	**(.025,.169)**	.028	(-.008,.070)
SDQ-Hyperactivity (Child)	**.151**	**(.128, .174)**	**.143**	**(.077,.208)**	.057	(-.002, .116)
Conners Total (Parent)	**.123**	**(.098,.148)**	**.062**	**(.002.127)**	.026	(-.011,.068)
Conners Hyperactivity-Impulsivity (Parent)	**.127**	**(.102,.155)**	**.079**	**(.015.150)**	.020	(-.016,.059)
Conners Inattention (Parent)	**.105**	**(.080,.130)**	.038	(-.022,.099)	.024	(-.016,.070)
SDQ-Conduct Problems (Parent)	**.107**	**(.083,.132)**	.050	(-.011,.116)	.004	(-.038,.052)
SDQ-Conduct Problems (Child)	**.144**	**(.119,.170)**	**.116**	**(.045,.187)**	.057	(-.005,.119)
SDQ-Prosocial (Parent)	.010	(-.011,.030)	.024	(-.031,.073)	.014	(-.014,.041)
SDQ-Prosocial (Child)	.005	(-.019,.029)	.014	(-.044,.073)	-.003	(-.063,.059)
SDQ-Peer Problems (Child)	**.119**	**(.094,.144)**	**.069**	**(.011.127)**	.029	(-.029,.091)
SPEQ-Paranoid Thoughts (Child)	**.173**	**(.147,.200)**	**.114**	**(.056,.174)**	**.059**	**(.003,.118)**
SPEQ-Hallucinations (Child)	**.135**	**(.111,.160)**	**.067**	**(.007,.126)**	.063	(-.002,.130)
SPEQ-Grandiosity (Child)	.013	(-.009,.037)	-.011	(-.070,.047)	-.007	(-.064.050)
SPEQ-Cognitive Disorganisation (Child)	**.153**	**(.130,.177)**	**.123**	**(.064,.181)**	**.092**	**(.037,.147)**
SPEQ-Anhedonia (Child)	**.060**	**(.037,.084)**	.016	(-.042,.076)	-.011	(-.074,.050)
SPEQ-Negative Symptoms (Parent)	**.056**	**(.034,.080)**	-.003	(-.056,.050)	-.013	(-.047,.019)

Note: Significant estimates are presented in **bold**. SDQ including Peer Problems refers to the four difficulty subscales of the SDQ: Anxiety, Hyperactivity, Conduct Problems and Peer Problems.

© 2017 Singham T et al. *JAMA Psychiatry*.

eTable 19. Two-Year Analyses With Social Victimization

Variable	Phenotypic		DZ Differences		MZ Differences	
	B	95% CI	B	95% CI	B	95% CI
SDQ Total Difficulties (Child)	**.238**	**(.206,.269)**	**.169**	**(.087,.256)**	**.112**	**(.044,.179)**
SDQ including Peer Problems (Child)	**.268**	**(.236,.301)**	**.188**	**(.104,.277)**	**.122**	**(.058,.190)**
ARBQ-Anxiety (Parent)	**.091**	**(.061,.123)**	.027	(-.032,.088)	.034	(-.018,.093)
CASI-Anxiety (Child)	**.192**	**(.160,.225)**	**.137**	**(.062,.215)**	**.117**	**(.050,.184)**
SDQ-Anxiety (Child)	**.186**	**(.155,.216)**	**.071**	**(-.002,.148)**	**.135**	**(.072,.196)**
MFQ-Depression (Parent)	**.129**	**(.096,.166)**	.062	(-.009,.129)	-.009	(-.078,.060)
MFQ-Depression (Child)	**.210**	**(.175,.245)**	**.168**	**(.098,.239)**	**.090**	**(.014,.163)**
SDQ-Hyperactivity (Parent)	**.105**	**(.076,.135)**	**.097**	**(.021,.176)**	.032	(-.019,.086)
SDQ-Hyperactivity (Child)	**.163**	**(.133, .194)**	**.144**	**(.055, .233)**	.059	(-.014, .131)
Conners Total (Parent)	**.112**	**(.081,.143)**	**.089**	**(.026,.150)**	.028	(-.015,.083)
Conners Hyperactivity-Impulsivity (Parent)	**.102**	**(.071,.135)**	.059	(-.004,.122)	.003	(-.039,.052)
Conners Inattention (Parent)	**.103**	**(.072,.134)**	**.092**	**(.023,.161)**	.038	(-.009,.096)
SDQ-Conduct Problems (Parent)	**.124**	**(.093,.155)**	**.111**	**(.043,.180)**	.013	(-.033,.063)
SDQ-Conduct Problems (Child)	**.186**	**(.153,.221)**	**.178**	**(.075,.280)**	.034	(-.048,.113)
SDQ-Prosocial (Parent)	-.003	(-.028,.023)	.023	(-.034,.078)	-.008	(-.042,.025)
SDQ-Prosocial (Child)	**.031**	**(.002,.060)**	.035	(-.041,.112)	-.051	(-.122,.021)
SDQ-Peer Problems (Child)	**.216**	**(.182,.249)**	**.142**	**(.049,.233)**	**.084**	**(.016,.156)**
SPEQ-Paranoid Thoughts (Child)	**.290**	**(.257,.325)**	**.210**	**(.137,.285)**	**.187**	**(.113,.261)**
SPEQ-Hallucinations (Child)	**.204**	**(.170,.239)**	**.124**	**(.043,.206)**	**.125**	**(.052,.205)**
SPEQ-Grandiosity (Child)	**.038**	**(.007,.070)**	**.089**	**(.006,.171)**	-.015	(-.081,.051)
SPEQ-Cognitive Disorganisation (Child)	**.224**	**(.195,.255)**	**.179**	**(.104,.255)**	**.126**	**(.063,.189)**
SPEQ-Anhedonia (Child)	**.096**	**(.065,.128)**	.037	(-.044,.116)	.045	(-.025,.114)
SPEQ-Negative Symptoms (Parent)	**.040**	**(.010,.071)**	-.018	(-.085,.047)	-.013	(-.050,.024)

Note: Significant estimates are presented in **bold**. SDQ including Peer Problems refers to the four difficulty subscales of the SDQ: Anxiety, Hyperactivity, Conduct Problems and Peer Problems.

© 2017 Singham T et al. *JAMA Psychiatry*.

eTable 20. Concurrent Analyses With Property-Related Victimization

Variable	Phenotypic		DZ Differences		MZ Differences	
	B	95% CI	B	95% CI	B	95% CI
SDQ Total Difficulties (Parent)	**.149**	**(.130,.170)**	**.085**	**(.038,.130)**	**.030**	**(.001,.060)**
SDQ Total Difficulties (Teacher)	**.130**	**(.104,.156)**	**.068**	**(.010,.129)**	**.052**	**(.002,.103)**
SDQ Total Difficulties (Child)	**.281**	**(.260,.301)**	**.184**	**(.134,.234)**	**.128**	**(.079,.177)**
SDQ including Peer Problems (Parent)	**.172**	**(.152,.193)**	**.108**	**(.065,.152)**	**.044**	**(.015,.074)**
SDQ including Peer Problems (Teacher)	**.143**	**(.117,.169)**	**.077**	**(.021,.134)**	**.064**	**(.016,.112)**
SDQ including Peer Problems (Child)	**.318**	**(.298,.339)**	**.220**	**(.172,.269)**	**.143**	**(.094,.191)**
SDQ-Anxiety (Parent)	**.082**	**(.062,.103)**	**.065**	**(.018,.112)**	.026	(-.017,.067)
SDQ-Anxiety (Teacher)	**.085**	**(.060,.111)**	**.090**	**(.031,.150)**	.039	(-.022,.097)
SDQ-Anxiety (Child)	**.223**	**(.202,.244)**	**.170**	**(.119,.220)**	**.142**	**(.090.194)**
MFQ-Depression (Parent)	**.125**	**(.103,.150)**	**.096**	**(.045,.151)**	**.062**	**(.014,.107)**
MFQ-Depression (Child)	**.309**	**(.285,.334)**	**.243**	**(.187,.299)**	**.222**	**(.169,.277)**
SDQ-Hyperactivity (Parent)	**.153**	**(.133,.174)**	**.061**	**(.007,.114)**	.023	(-.005,.053)
SDQ-Hyperactivity (Teacher)	**.117**	**(.092,.143)**	.049	(-.011,.107)	**.058**	**(.012,.106)**
SDQ-Hyperactivity (Child)	**.198**	**(.178,.218)**	**.104**	**(.054,.154)**	**.054**	**(.008,.104)**
Conners Total (Parent)	**.159**	**(.138,.180)**	**.085**	**(.038,.136)**	.019	(-.006,.044)
Conners Hyperactivity-Impusivity (Parent)	**.121**	**(.100,.143)**	**.070**	**(.022,.119)**	.012	(-.010,.036)
Conners Inattention (Parent)	**.169**	**(.148,.190)**	**.083**	**(.036,.135)**	.022	(-.007,.054)
SDQ-Conduct Problems (Parent)	**.116**	**(.095,.138)**	**.064**	**(.015,.117)**	.017	(-.013,.048)
SDQ-Conduct Problems (Teacher)	**.083**	**(.057,.112)**	.002	(-.064,.066)	.005	(-.052,.063)
SDQ-Conduct Problems(Child)	**.245**	**(.224,.267)**	**.154**	**(.097,.207)**	**.104**	**(.054,.158)**
SDQ-Prosocial (Parent)	**-.033**	**(-.051,-.015)**	.011	(-.035,.057)	.000	(-.026,.027)
SDQ-Prosocial (Teacher)	**-.058**	**(-.081,-.034)**	-.004	(-.060,.051)	-.024	(-.069,.020)
SDQ-Prosocial (Child)	**-.022**	**(-.041,-.002)**	.017	(-.035,.069)	.018	(-.027,.061)
SDQ-Peer Problems (Parent)	**.175**	**(.151,.199)**	**.124**	**(.068,.177)**	**.065**	**(.027,.105)**
SDQ-Peer Problems (Teacher)	**.115**	**(.088,.142)**	**.066**	**(.010,.127)**	**.070**	**(.011,.124)**
SDQ-Peer Problems (Child)	**.288**	**(.264,.312)**	**.221**	**(.162,.279)**	**.117**	**(.057,.172)**

Note: Significant estimates are presented in **bold**. SDQ including Peer Problems refers to the four difficulty subscales of the SDQ: Anxiety, Hyperactivity, Conduct Problems and Peer Problems.

© 2017 Singham T et al. *JAMA Psychiatry*.

eTable 21. Five-Year Analyses With Property-Related Victimization

Variable	Phenotypic		DZ Differences		MZ Differences	
	B	95% CI	B	95% CI	B	95% CI
SDQ Total Difficulties (Child)	**.117**	**(.093,.141)**	.052	(-.008,.111)	.000	(-.052,.050)
SDQ including Peer Problems (Child)	**.132**	**(.108,.156)**	.056	(-.004,.114)	.004	(-.048,.056)
ARBQ-Anxiety (Parent)	**.029**	**(.005,.053)**	.031	(-.018,.092)	-.001	(-.061,.047)
CASI-Anxiety (Child)	**.064**	**(.040,.090)**	**.088**	**(.033,.144)**	.016	(-.032,.067)
SDQ-Anxiety (Child)	**.042**	**(.017,.066)**	.038	(-.022,.094)	.003	(-.046,.054)
MFQ-Depression (Parent)	**.060**	**(.035,.087)**	.040	(-.015,.105)	-.052	(-.141,.003)
MFQ-Depression (Child)	**.090**	**(.065,.117)**	.043	(-.021,.104)	-.019	(-.079,.039)
SDQ-Hyperactivity (Parent)	**.120**	**(.096,.145)**	**.077**	**(.011,.145)**	.002	(-.038,.043)
SDQ-Hyperactivity (Child)	**0.123**	**(.100, .146)**	.039	(-.022, .100)	.011	(-.046,.070)
Conners Total (Parent)	**.125**	**(.098,.151)**	**.060**	**(.000,.126)**	.037	(.002,.078)
Conners Hyperactivity-Impulsivity (Parent)	**.089**	**(.062,.118)**	.064	(-.004,.138)	.020	(-.018,.059)
Conners Inattention (Parent)	**.130**	**(.104,.157)**	.045	(-.019,.111)	**.040**	**(.003,.086)**
SDQ-Conduct Problems (Parent)	**.074**	**(.048,.100)**	.027	(-.031,.092)	-.004	(-.046,.038)
SDQ-Conduct Problems (Child)	**.119**	**(.093,.144)**	.036	(-.033,.104)	-.023	(-.083,.035)
SDQ-Prosocial (Parent)	**-.024**	**(-.045,-.004)**	-.009	(-.063,.043)	.012	(-.011,.035)
SDQ-Prosocial (Child)	**-.046**	**(-.070,-.022)**	.011	(-.048,.071)	-.006	(-.061,.048)
SDQ-Peer Problems (Child)	**.114**	**(.089,.140)**	.038	(-.021,.097)	.018	(-.040,.074)
SPEQ-Paranoid Thoughts (Child)	**.149**	**(.124,.176)**	**.068**	**(.008,.125)**	.046	(-.006,.099)
SPEQ-Hallucinations (Child)	**.117**	**(.092,.144)**	.057	(-.010,.120)	.050	(-.007,.112)
SPEQ-Grandiosity (Child)	**.036**	**(.012,.061)**	-.007	(-.067,.052)	-.011	(-.063,.043)
SPEQ-Cognitive Disorganisation (Child)	**.090**	**(.067,.113)**	.050	(-.006,.108)	.015	(-.039,.073)
SPEQ-Anhedonia (Child)	**.088**	**(.065,.112)**	.022	(-.037,.079)	.029	(-.033,.089)
SPEQ-Negative Symptoms (Parent)	**.072**	**(.047,.098)**	.030	(-.036,.096)	.025	(-.013,.057)

Note: Significant estimates are presented in **bold**. SDQ including Peer Problems refers to the four difficulty subscales of the SDQ: Anxiety, Hyperactivity, Conduct Problems and Peer Problems.

© 2017 Singham T et al. *JAMA Psychiatry.*

eTable 22. Two-Year Analyses With Property-Related Victimization

Variable	Phenotypic		DZ Differences		MZ Differences	
	B	95% CI	B	95% CI	B	95% CI
SDQ Total Difficulties (Child)	**.148**	**(.115,.180)**	**.154**	**(.074,.240)**	.021	(-.047,.088)
SDQ including Peer Problems (Child)	**.185**	**(.153,.217)**	**.177**	**(.099,.261)**	.016	(-.048,.081)
ARBQ-Anxiety (Parent)	**.056**	**(.024,.089)**	**.057**	**(-.004,.128)**	**.067**	**(.013,.137)**
CASI-Anxiety (Child)	**.071**	**(.039,.105)**	**.091**	**(.023,.161)**	.030	(-.034,.097)
SDQ-Anxiety (Child)	**.070**	**(.039,.100)**	**.074**	**(.003,.147)**	.028	(-.034,.094)
MFQ-Depression (Parent)	**.086**	**(.053,.123)**	.059	(-.025,.138)	.035	(-.026,.095)
MFQ-Depression (Child)	**.114**	**(.081,.148)**	.064	(-.012,.146)	.012	(-.062,.085)
SDQ-Hyperactivity (Parent)	**.132**	**(.101,.165)**	.078	(-.006,.158)	.036	(-.016,.106)
SDQ-Hyperactivity (Child)	**.133**	**(.101, .163)**	**.143**	**(062, .229)**	.013	(-.056, 082)
Conners Total (Parent)	**.131**	**(.097,.167)**	.043	(-.033,.104)	.022	(-.026,.119)
Conners Hyperactivity-Impulsivity (Parent)	**.083**	**(.050,.121)**	-.007	(-.079,.061)	.010	(-.035,.093)
Conners Inattention (Parent)	**.139**	**(.105,.176)**	.068	(-.012,.138)	.026	(-.023,.115)
SDQ-Conduct Problems (Parent)	**.082**	**(.051,.115)**	.050	(-.027,.120)	-.006	(-.051,.039)
SDQ-Conduct Problems (Child)	**.140**	**(.107,.175)**	**.125**	**(.026,.220)**	-.001	(-.081,.075)
SDQ-Prosocial (Parent)	**-.051**	**(-.079,-.025)**	.014	(-.040,.077)	-.016	(-.052,.020)
SDQ-Prosocial (Child)	**-.057**	**(-.088,-.026)**	-.017	(-.085,.049)	-.020	(-.100,.061)
SDQ-Peer Problems (Child)	**.200**	**(.166,.235)**	**.149**	**(.061,.237)**	-.010	(-.080,.056)
SPEQ-Paranoid Thoughts (Child)	**.240**	**(.206,.276)**	**.179**	**(.112,.254)**	**.093**	**(.022,.168)**
SPEQ-Hallucinations (Child)	**.149**	**(.114,.185)**	**.103**	**(.029,.179)**	.069	(-.008,.150)
SPEQ-Grandiosity (Child)	**.043**	**(.012,.073)**	.004	(-.079,.084)	-.046	(-.112,.018)
SPEQ-Cognitive Disorganisation (Child)	**.127**	**(.096,.157)**	**.160**	**(.090,.232)**	.061	(-.010,.130)
SPEQ-Anhedonia (Child)	**.132**	**(.102,.163)**	.074	(-.003,.146)	-.003	(-.082,.071)
SPEQ-Negative Symptoms (Parent)	**.079**	**(.048,.114)**	**.026**	**(-.035,.091)**	.021	(-.019,.071)

Note: Significant estimates are presented in **bold**. SDQ including Peer Problems refers to the four difficulty subscales of the SDQ: Anxiety, Hyperactivity, Conduct Problems and Peer Problems.

© 2017 Singham T et al. *JAMA Psychiatry.*

eTable 23. Intercorrelations of Victimization Subscales at Concurrent

	Physical	Verbal	Social	Property
Physical	(.81)			
Verbal	.63	(.82)		
Social	.51	.59	(.80)	
Property	.57	.55	.54	(.80)

Note: Cronbach's alpha for each subscale is given down the diagonal in parentheses

© 2017 Singham T et al. *JAMA Psychiatry*.

eTable 24. Intercorrelations of Victimization Subscales at Age 14 Years

	Physical	Verbal	Social	Property
Physical	(.81)			
Verbal	.53	(.84)		
Social	.42	.58	(.81)	
Property	.60	.54	.50	(.83)

Note: Cronbach's alpha for each subscale is given down the diagonal in parentheses

© 2017 Singham T et al. *JAMA Psychiatry*.

eTable 25. Moderation by Sex of Phenotypic Estimates, for Peer Victimization at Age 11 Years and Mental Health Outcomes at Age 11 Years (i.e. Concurrent Effect) and at Age 16 Years (i.e. Five-Year Effect)

Outcome	Timing	Variable Name	Male model			Female model			Comparison model fits		
			B	95% CI		B	95% CI		χ2 difference	*p*	*p* adjusted*
Total Difficulties	Concurrent	SDQ Total Difficulties (Parent)	.211	.183	.239	.251	.222	.281	3.859	.049	.212
		SDQ Total Difficulties (Teacher)	.205	.164	.245	.195	.158	.231	.130	.719	1.000
		SDQ Total Difficulties (Child)	.377	.350	.404	.425	.397	.453	5.777	.016	.089
	Five-year	SDQ Total Difficulties (Child)	.152	.118	.186	.218	.184	.252	7.248	.007	.050
Total Difficulties (Including peer problems)	Concurrent	SDQ including Peer Problems (Parent)	.255	.226	.283	.282	.252	.312	1.684	.194	.484
		SDQ including Peer Problems (Teacher)	.234	.193	.275	.193	.156	.229	2.196	.138	.432
		SDQ including Peer Problems (Child)	.426	.399	.453	.487	.459	.515	9.453	.002	.024
	Five-year	SDQ including Peer Problems (Child)	.174	.140	.207	.244	.209	.278	8.033	.005	.040
Anxiety & Depression	Concurrent	SDQ-Anxiety (Parent)	.114	.087	.141	.168	.138	.199	6.654	.010	.060
		SDQ-Anxiety (Teacher)	.164	.126	.203	.129	.091	.167	1.677	.195	.484
		SDQ-Anxiety (Child)	.291	.264	.318	.380	.349	.411	17.849	.000	.000
		MFQ-Depression (Parent)	.171	.140	.201	.218	.182	.254	3.850	.050	.212
		MFQ-Depression (Child)	.388	.357	.419	.472	.436	.508	12.321	.000	.000
	Five-year	SDQ-Anxiety (Child)	.044	.013	.074	.136	.100	.172	14.684	.000	.000
		ARBQ-Anxiety (Parent)	.074	.041	.108	.058	.023	.093	.460	.498	.875
		CASI-Anxiety (Child)	.079	.047	.110	.149	.112	.186	8.001	.005	.040
		MFQ-Depression (Parent)	.092	.058	.125	.114	.073	.155	.702	.402	.751
		MFQ-Depression (Child)	.106	.075	.136	.160	.121	.200	4.505	.034	.173
Hyper-activity / Inattention / Impulsiveness	Concurrent	SDQ-Hyperactivity (Parent)	.198	.169	.227	.227	.198	.256	1.883	.170	.484
		SDQ-Hyperactivity (Teacher)	.174	.136	.212	.142	.108	.175	1.564	.211	.506
		SDQ-Hyperactivity (Child)	.258	.231	.285	.275	.248	.302	.731	.392	.751
		Conners Total (Parent)	.223	.192	.254	.230	.201	.260	.112	.738	1.000
		Conners Hyp-Impul (Parent)	.197	.166	.229	.179	.150	.208	.688	.407	.751
		Conners Inatt (Parent)	.209	.180	.238	.237	.207	.267	1.688	.194	.484
	Five-year	SDQ-Hyperactivity (Parent)	.175	.139	.212	.172	.135	.208	.021	.886	1.000
		SDQ-Hyperactivity (Child)	.149	.116	.182	.198	.166	.230	4.380	.036	.173
		Conners Total (Parent)	.222	.181	.263	.148	.115	.182	7.749	.005	.040
		Conners Hyp-Impul (Parent)	.158	.117	.198	.143	.104	.182	.270	.603	1.000
		Conners Inatt (Parent)	.213	.173	.252	.145	.112	.177	7.011	.008	.052
Conduct Problems	Concurrent	SDQ-CP (Parent)	.165	.135	.195	.200	.169	.231	2.589	.108	.389
		SDQ-CP (Teacher)	.136	.093	.179	.129	.091	.166	.059	.809	1.000
		SDQ-CP(Child)	.328	.298	.357	.351	.323	.379	1.233	.267	.580
	Five-year	SDQ-CP (Parent)	.153	.115	.190	.117	.083	.152	1.811	.178	.484
		SDQ-CP (Child)	.158	.122	.193	.189	.152	.225	1.424	.233	.541
Pro-social	Concurrent	SDQ-Prosocial (Parent)	.011	-.015	.037	-.060	-.086	-.033	14.436	.000	.000
		SDQ-Prosocial (Teacher)	-.078	-.113	-.043	-.051	-.085	-.017	1.195	.274	.580
		SDQ-Prosocial (Child)	.021	-.007	.049	-.052	-.079	-.025	13.998	.000	.000
	Five-year	SDQ-Prosocial (Parent)	-.027	-.060	.006	-.024	-.054	.006	.021	.885	1.000
		SDQ-Prosocial (Child)	-.037	-.071	-.002	-.059	-.092	-.025	.809	.369	.738

© 2017 Singham T et al. *JAMA Psychiatry*.

eTable 25. Moderation by Sex of Phenotypic Estimates, for Peer Victimization at Age 11 Years and Mental Health Outcomes at Age 16 Years (i.e. Concurrent Effect) and at Age 16 Years (i.e. Five-Year Effect)

Peer Problems	Concurrent	SDQ-Peer Problems (Parent)	.266	.232	.301		.265	.232	.298	.004	.948	1.000
		SDQ-Peer Problems (Teacher)	.189	.148	.231		.151	.111	.190	1.738	.187	.484
		SDQ-Peer Problems (Child)	.388	.356	.421	z	.428	.394	.462	2.678	.102	.387
	Five-year	SDQ-Peer Problems (Child)	.154	.118	.189		.180	.143	.216	.993	.319	.656
Psychotic-like Symptoms	Five-year	SPEQ-Paranoid Thoughts (Child)	.191	.155	.228		.233	.195	.271	2.442	.118	.405
		SPEQ-Hallucinations (Child)	.123	.088	.159		.171	.136	.207	3.397	.065	.260
		SPEQ-Grandiosity (Child)	.056	.020	.091		.018	-.015	.050	2.366	.124	.406
		SPEQ-Cognitive Disorganisation (Child)	.135	.102	.169		.162	.128	.196	1.194	.274	.580
		SPEQ-Anhedonia (Child)	.095	.061	.129		.114	.081	.147	.613	.434	.781
		SPEQ-Negative Symptoms (Parent)	.092	.056	.127		.098	.062	.135	.062	.803	1.000

Note: Sex differences in the effects of victimization on mental health outcome were tested by estimating two phenotypic models: 1) *unconstrained* model (i.e. parameters allowed to differ between sexes); 2) *constrained* model: regression parameter reflecting the association between mental health and victimization allowed to differ by sex. The two models were then estimated using a Maximum Likelihood Robust estimator and compared using a scaled difference chi-square test statistics, following previous research [3], implemented in the SEM software *Lavaan.*
* adjusted p-value estimates using the false discovery rate procedure to control for multiple comparisons (k=72)

© 2017 Singham T et al. *JAMA Psychiatry.*

eTable 26. Moderation by Sex of Phenotypic Estimates: Same-Sex Twin Differences for Peer Victimization at Age 14 Years and Mental Health Outcomes at Age 16 Years (i.e. Two-Year Analyses)

Outcome	Timing	Variable Name	Male model			Female model			Comparison model fits		
			B	95% CI		B	95% CI		χ2 difference	*p*	*p* adjusted*
Total Difficulties	Two-year	SDQ Total Difficulties (Child)	.228	.181	.275	.270	.226	.315	1.684	.194	1.000
Total Difficulties (Including peer problems)	Two-year	SDQ including Peer Problems (Child)	.261	.215	.306	.329	.283	.375	4.291	.038	.547
Anxiety & Depression	Two-year	SDQ-Anxiety (Child)	.102	.061	.143	.199	.152	.245	9.352	.002	.144
		ARBQ-Anxiety (Parent)	.092	.050	.133	.081	.031	.131	.106	.745	1.000
		CASI-Anxiety (Child)	.100	.059	.142	.196	.147	.245	8.355	.004	.144
		MFQ-Depression (Parent)	.102	.062	.142	.168	.109	.227	3.550	.060	.627
		MFQ-Depression (Child)	.157	.114	.200	.246	.193	.299	6.468	.011	.198
Hyper-activity / Inattention / Impulsiveness	Two-year	SDQ-Hyperactivity (Parent)	.163	.120	.206	.172	.126	.218	.067	.796	1.000
		SDQ-Hyperactivity (Child)	.187	.142	.231	.208	.166	.251	.489	.484	1.000
		Conners Total (Parent)	.172	.127	.216	.194	.140	.248	.391	.532	1.000
		Conners Hyp-Impul (Parent)	.135	.085	.185	.134	.081	.187	.001	.979	1.000
		Conners Inatt (Parent)	.163	.120	.207	.200	.147	.252	1.125	.289	1.000
Conduct Problems	Two-year	SDQ-CP (Parent)	.148	.105	.191	.159	.110	.208	.102	.749	1.000
		SDQ-CP (Child)	.238	.190	.287	.203	.157	.248	1.100	.294	1.000
Pro-social	Two-year	SDQ-Prosocial (Parent)	-.046	-.083	-.009	-.065	-.103	-.028	.514	.474	1.000
		SDQ-Prosocial (Child)	-.049	-.093	-.005	-.044	-.086	-.003	.020	.888	1.000
Peer Problems	Two-year	SDQ-Peer Problems (Child)	.256	.207	.305	.278	.231	.324	.388	.534	1.000
Psychotic-like Symptoms	Two-year	SPEQ-Paranoid Thoughts (Child)	.330	.283	.378	.366	.317	.416	1.052	.305	1.000
		SPEQ-Hallucinations (Child)	.171	.128	.214	.266	.213	.320	7.342	.007	.168
		SPEQ-Grandiosity (Child)	.047	.005	.090	.049	.004	.095	.004	.949	1.000
		SPEQ-Cognitive Disorganisation (Child)	.184	.143	.224	.240	.197	.284	3.512	.061	.627
		SPEQ-Anhedonia (Child)	.157	.113	.202	.132	.090	.175	.634	.426	1.000
		SPEQ-Negative Symptoms (Parent)	.074	.036	.112	.109	.055	.164	1.115	.291	1.000

Note: See eTable 25.

© 2017 Singham T et al. *JAMA Psychiatry*.

eTable 27. Linear and Quadratic Effects in Phenotypic Analyses, Peer Victimization at Age 11 Years and Mental Health Outcomes at Age 11 Years (i.e. Concurrent Effect) and at Age 16 Years (i.e. Five-Year Effect)

Outcome	Timing	Variable Name	Linear effects			Nonlinear effects		
			B	95% CI		B	95% CI	
Total Difficulties	Concurrent	SDQ Total Difficulties (Parent)	**.247**	**.223**	**.271**	-.013	-.029	.005
		SDQ Total Difficulties (Teacher)	**.205**	**.175**	**.237**	.002	-.020	.025
		SDQ Total Difficulties (Child)	**.439**	**.416**	**.463**	**-.035**	**-.050**	**-.019**
	Five-year	SDQ Total Difficulties (Child)	**.235**	**.205**	**.266**	**-.050**	**-.070**	**-.031**
Total Difficulties (Including peer problems)	Concurrent	SDQ including Peer Problems (Parent)	**.275**	**.251**	**.299**	-.004	-.021	.013
		SDQ including Peer Problems (Teacher)	**.217**	**.187**	**.248**	.006	-.017	.030
		SDQ including Peer Problems (Child)	**.481**	**.457**	**.503**	**-.022**	**-.037**	**-.006**
	Five-year	SDQ including Peer Problems (Child)	**.260**	**.229**	**.290**	**-.051**	**-.071**	**-.032**
Anxiety & Depression	Concurrent	SDQ-Anxiety (Parent)	**.167**	**.142**	**.193**	**-.028**	**-.045**	**-.012**
		SDQ-Anxiety (Teacher)	**.168**	**.135**	**.200**	-.019	-.042	.004
		SDQ-Anxiety (Child)	**.359**	**.334**	**.384**	**-.033**	**-.050**	**-.016**
		MFQ-Depression (Parent)	**.196**	**.171**	**.221**	-.003	-.023	.016
		MFQ-Depression (Child)	**.435**	**.411**	**.460**	-.008	-.028	.012
	Five-year	SDQ-Anxiety (Child)	**.137**	**.106**	**.170**	**-.058**	**-.078**	**-.039**
		ARBQ-Anxiety (Parent)	**.074**	**.045**	**.105**	-.014	-.033	.007
		CASI-Anxiety (Child)	**.144**	**.113**	**.176**	**-.042**	**-.062**	**-.022**
		MFQ-Depression (Parent)	**.127**	**.094**	**.158**	**-.027**	**-.049**	**-.002**
		MFQ-Depression (Child)	**.180**	**.150**	**.214**	**-.050**	**-.071**	**-.029**
Hyper-activity / Inattention / Impulsiveness	Concurrent	SDQ-Hyperactivity (Parent)	**.228**	**.203**	**.254**	-.009	-.026	.009
		SDQ-Hyperactivity (Teacher)	**.165**	**.135**	**.196**	.006	-.015	.027
		SDQ-Hyperactivity (Child)	**.302**	**.277**	**.327**	**-.028**	**-.044**	**-.012**
		Conners Total (Parent)	**.224**	**.200**	**.249**	.006	-.012	.024
		Conners Hyp-Impul (Parent)	**.180**	**.156**	**.204**	.011	-.008	.030
		Conners Inatt (Parent)	**.228**	**.202**	**.253**	-.001	-.017	.018
	Five-year	SDQ-Hyperactivity (Parent)	**.197**	**.166**	**.228**	-.016	-.038	.006
		SDQ-Hyperactivity (Child)	**.203**	**.172**	**.233**	**-.027**	**-.046**	**-.008**
		Conners Total (Parent)	**.205**	**.174**	**.235**	-.014	-.035	.009
		Conners Hyp-Impul (Parent)	**.139**	**.109**	**.169**	.008	-.015	.034
		Conners Inatt (Parent)	**.216**	**.186**	**.248**	**-.028**	**-.049**	**-.005**
Conduct Problems	Concurrent	SDQ-CP (Parent)	**.176**	**.152**	**.200**	.007	-.010	.025
		SDQ-CP (Teacher)	**.118**	**.087**	**.149**	.020	-.003	.044
		SDQ-CP(Child)	**.365**	**.341**	**.389**	**-.020**	**-.036**	**-.002**
	Five-year	SDQ-CP (Parent)	**.144**	**.114**	**.175**	-.009	-.030	.014
		SDQ-CP (Child)	**.198**	**.166**	**.229**	-.021	-.042	.001
Pro-social	Concurrent	SDQ-Prosocial (Parent)	**-.038**	**-.063**	**-.014**	.009	-.006	.024
		SDQ-Prosocial (Teacher)	**-.063**	**-.093**	**-.030**	-.011	-.031	.008

© 2017 Singham T et al. *JAMA Psychiatry*.

eTable 27. Linear and Quadratic Effects in Phenotypic Analyses, Peer Victimization at Age 11 Years and Mental Health Outcomes at Age 11 Years (i.e. Concurrent Effect) [illegible]

		SDQ-Prosocial (Child)	**-.061**	**-.087**	**-.034**	**.032**	**.016**	**.049**
	Five-year	SDQ-Prosocial (Parent)	**-.034**	**-.063**	**-.005**	.004	-.013	.020
		SDQ-Prosocial (Child)	**-.073**	**-.106**	**-.042**	.011	-.010	.031
Peer Problems	Concurrent	SDQ-Peer Problems (Parent)	**.244**	**.219**	**.269**	**.021**	**.001**	**.042**
		SDQ-Peer Problems (Teacher)	**.160**	**.129**	**.190**	.014	-.010	.039
		SDQ-Peer Problems (Child)	**.380**	**.354**	**.404**	**.026**	**.006**	**.046**
	Five-year	SDQ-Peer Problems (Child)	**.197**	**.166**	**.229**	**-.026**	**-.046**	**-.004**
Psychotic-like Symptoms	Five-year	SPEQ-Paranoid Thoughts (Child)	**.250**	**.220**	**.282**	**-.037**	**-.058**	**-.016**
		SPEQ-Hallucinations (Child)	**.189**	**.157**	**.221**	**-.038**	**-.059**	**-.017**
		SPEQ-Grandiosity (Child)	**.050**	**.019**	**.083**	-.006	-.026	.015
		SPEQ-Cognitive Disorganisation (Child)	**.197**	**.165**	**.228**	**-.051**	**-.070**	**-.031**
		SPEQ-Anhedonia (Child)	**.128**	**.096**	**.159**	-.015	-.034	.004
		SPEQ-Negative Symptoms (Parent)	**.109**	**.078**	**.141**	-.011	-.033	.012

Note: The phenotypic models reported in the main manuscript tested a linear relationship between peer victimization and mental health. A plausible hypothesis of a non-linear relationship is that peer victimization has increasing impact at higher levels of exposure. We tested such possible nonlinear relationships by adding quadratic terms into the phenotypic models and bootstrapping the estimates. According to the aforementioned hypothesis, we would expect positive quadratic terms (i.e. flat or low effects at low scores of victimization and more rapidly increasing effects at higher scores of victimization). All estimates were bootstrapped. Significant estimates are presented in **bold.**

© 2017 Singham T et al. *JAMA Psychiatry*.

eReferences

1. Dodge KA, Pettit GS, Bates JE. Socialization mediators of the relation between socioeconomic status and child conduct problems. *Child development.* 1994;65(2):649-665.
2. Deater-Deckard K. The Parent Feelings Questionnaire. *London: Institute of Psychiatry.* 1996.
3. Satorra A, Bentler PM. A scaled difference chi-square test statistic for moment structure analysis. *Psychometrika.* 2001;66(4):507-514.

© 2017 Singham T et al. *JAMA Psychiatry*.
